# Autophagy in spinal muscular atrophy: from pathogenic mechanisms to therapeutic approaches

**DOI:** 10.3389/fncel.2023.1307636

**Published:** 2024-01-08

**Authors:** Saman Rashid, Maria Dimitriadi

**Affiliations:** School of Life and Medical Science, University of Hertfordshire, Hatfield, United Kingdom

**Keywords:** spinal muscular atrophy, autophagy, macroautophagy, mitophagy, autophagic flux

## Abstract

Spinal muscular atrophy (SMA) is a devastating neuromuscular disorder caused by the depletion of the ubiquitously expressed survival motor neuron (SMN) protein. While the genetic cause of SMA has been well documented, the exact mechanism(s) by which SMN depletion results in disease progression remain elusive. A wide body of evidence has highlighted the involvement and dysregulation of autophagy in SMA. Autophagy is a highly conserved lysosomal degradation process which is necessary for cellular homeostasis; defects in the autophagic machinery have been linked with a wide range of neurodegenerative disorders, including amyotrophic lateral sclerosis, Alzheimer’s disease and Parkinson’s disease. The pathway is particularly known to prevent neurodegeneration and has been suggested to act as a neuroprotective factor, thus presenting an attractive target for novel therapies for SMA patients. In this review, (a) we provide for the first time a comprehensive summary of the perturbations in the autophagic networks that characterize SMA development, (b) highlight the autophagic regulators which may play a key role in SMA pathogenesis and (c) propose decreased autophagic flux as the causative agent underlying the autophagic dysregulation observed in these patients.

## Spinal muscular atrophy

**FIGURE 1:**
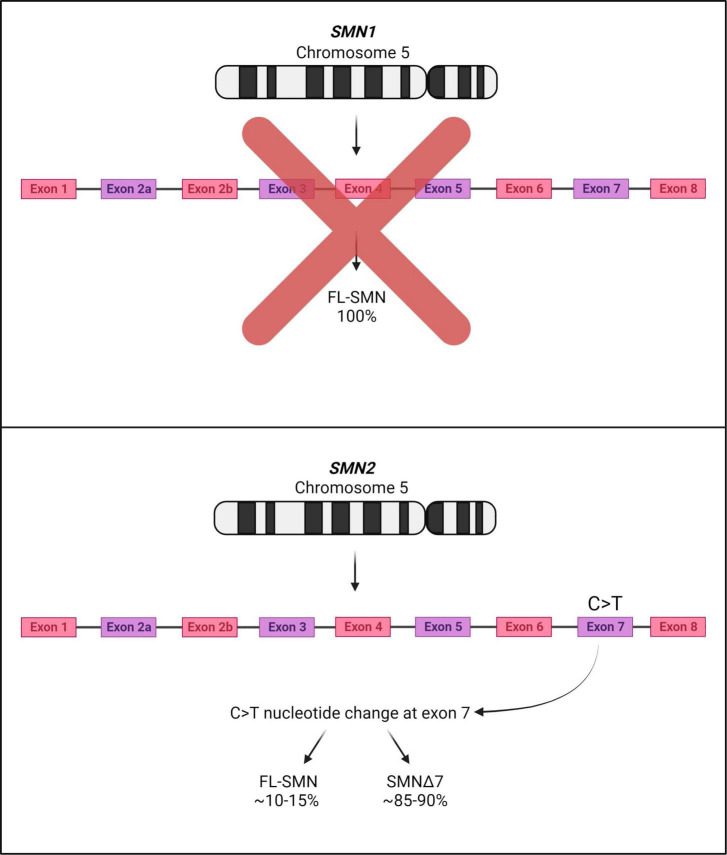
SMA associated genes *SMN1* and *SMN2* on human chromosome 5. SMA patients display deletions or mutations in both copies of *SMN1*; although *SMN2* is expressed, most of the *SMN2* mRNA transcripts lack exon 7 due to a C > T nucleotide change. Thus, most of the product deriving from the *SMN2* gene is mostly unstable and rapidly degraded (SMNΔ7).

Spinal muscular atrophy (SMA) is a devastating autosomal recessive neuromuscular disorder, presenting with an overall incidence rate of 1 in 6,000 to 1 in 10,000 live births and a carrier frequency of around 1 in 40 Caucasian adults (Crawford and Pardo, 1996; Verhaart et al., 2017). It is caused by the reduced expression of the ubiquitous survival motor neuron (SMN) protein, resulting in (a) degeneration of the α-motor neurons in the anterior horn of the spinal cord, (b) neuromuscular junction (NMJ) maturation defects, (c) progressive muscle atrophy and (d) ultimately death (Monani and De Vivo, 2014). It is now well-documented that two genes code for the SMN protein in humans: the telomeric *survival motor neuron 1* (*SMN1*) and the centromeric *survival motor neuron 2* (*SMN2*), both located on chromosome 5q13. Heterozygous carriers of the disease harbor a single copy of *SMN1*, however due to a gene duplication event, multiple copies of *SMN2* can be presented (Lorson et al., 1999; Monani et al., 1999). The *SMN1* gene codes for a fully functional SMN protein, while a single C > T nucleotide change in *SMN2* disrupts an exonic splicing enhancer, a DNA sequence motif which promotes the inclusion of exons into mature transcripts. This genetic change instead creates a new exonic splicing silencer that results in exon 7 skipping and the formation of a truncated variant of the SMN protein - known as SMNΔ7 with diminished function and stability (Lorson et al., 1999; Cartegni and Krainer, 2002; Kashima and Manley, 2003; Figure 1). Approximately 85–90% of the *SMN2* gene product accounts for SMNΔ7, while 10–15% codes for a functional SMN protein (Kolb and Kissel, 2015). The latter is not adequate to prevent SMA; although, it is of note that more copies of *SMN2* result in less severe forms of the disease (Wirth, 2021).

### Clinical presentations of SMA

Spinal muscular atrophy is typically categorized as proximal and distal SMA. Proximal SMA is the most common form of the disease, accounting for up to 95% of cases and manifests during infancy or early childhood, affecting the proximal regions. Conversely, distal SMA is significantly less common and occurs during childhood but progresses at a slower rate into adulthood (Farrar and Kiernan, 2015). SMA is clinically divided into four types, types I-IV with type 0 referring to prenatal SMA. Diagnosis of disease depends on the severity, age of onset and motor function. Type 0 SMA refers to neonates with one copy of *SMN2*, displaying severe weakness and a history of decreased fetal movements *in utero* (MacLeod et al., 1999). Type I SMA, which is often referred to as Werdnig-Hoffmann disease, manifests within 6 months of age and is characterized by hypotonia, poor head control and an inability to sit unaided. Type I SMA patients have two copies of *SMN2* and apart from poor motor neuron function, they usually develop respiratory complications within 2 years of symptom onset (Thomas and Dubowitz, 1994; Zerres and Rudnik-Schöneborn, 1995; Finkel et al., 2014). In contrast, children with three copies of *SMN2* are categorized under type II SMA (an intermediate form of disease), can sit without aid during their development and display independent head control present with limited hypotonia. SMA type III subjects have three or four *SMN2* copies and are known to walk independently at some point in their lives, although patients present with a progressive weakness in leg muscles. Life expectancy of patients with type III SMA is not affected (Zerres et al., 1997). Patients with more than four copies of *SMN2* are characterized by type IV SMA, which is the mildest form of the disease, making up less than 5% of all cases. Onset of type IV is typically during adulthood and is characterized by mild muscle weakness and reduced fine motor control (Zerres and Rudnik-Schöneborn, 1995).

### Molecular mechanisms underlying SMA

Various *in vitro* and *in vivo* models have been used to understand the molecular and cellular mechanisms underlying SMA pathogenesis (Monani et al., 2000; Edens et al., 2015; O’Hern et al., 2017; Nishio et al., 2023). Although a detailed report on SMA pathogenesis is beyond the scope of this review, a few important aspects are discussed below.

Survival motor neuron is expressed in the nucleus and cytoplasm. Its canonical function is the assembly of small nuclear ribonucleoproteins (snRNPs), which are critical for pre-mRNA splicing, through interactions with the Gemin complex (Fischer et al., 1997). Hence, it is of note that splicing defects are described in many aspects of SMA pathology (Burghes and Beattie, 2009; Singh and Singh, 2018). Besides SMN’s canonical role, the protein is also involved in RNA transport and local translation in axons, an event particularly important in neuronal cells for proper neuronal development (Fallini et al., 2012). SMN binds RNA via a domain implicated in the localization of β-actin mRNA to the axonal growth cones of motor neurons, a process necessary for correct axonal development (Rossoll et al., 2003). Furthermore, SMN is known to interact with proteins involved in cytoskeletal dynamics and it has been observed that granules containing SMN are associated with cytoskeletal microfilaments essential for cell shape, integrity and transport along the axonal compartment in neurons (Béchade et al., 1999). Thus, the interaction of SMN with β-actin further highlights the integral role of SMN in cytoskeletal and microtubule dynamics. Interestingly, SMN also binds profilin2a, a neuron specific actin-binding protein (Giesemann et al., 1999). Profilin2a is regulated by Rho-Associated Kinase (ROCK) and ROCK inhibition displayed increased lifespan and amelioration of defects at the NMJ of intermediate SMA mice (Bowerman et al., 2010, 2012). SMN protein has also been implicated in improper proteostasis (Zhang et al., 2013; Lambert-Smith et al., 2020). Proteomic studies revealed SMN depletion resulted in reduced expression of ubiquitin-like modifier activating enzyme 1 (UBA1) – a highly conserved protein ubiquitously expressed throughout all human tissues, canonically tasked with a role in the unfolded protein response and autophagy (Chang et al., 2013; Lambert-Smith et al., 2020). Reduced UBA1 resulted in disrupted proteosome degradation and perturbed axonal morphology (Hosseinibarkooie et al., 2017), whereas restoration of UBA1 mRNA rescued functional and morphological defects in SMA zebrafish (Powis et al., 2016). Furthermore, a growing body of evidence has also highlighted the role of SMN in endocytosis (Dimitriadi et al., 2010; Hosseinibarkooie et al., 2017; Riessland et al., 2017). Given the dependence of endocytosis on cytoskeletal remodeling (Montagnac et al., 2013; Franck et al., 2019), involvement of the SMN protein in endocytosis is not a surprise. Research on mammalian and nematode SMA models demonstrated perturbations in the endocytic pathway. In the nematode *Caenorhabditis elegans*, SMN ortholog depletion impaired synaptic transmission by interfering with the recycling of endocytic synaptic vesicles (Dimitriadi et al., 2010). Furthermore, diminished levels of SMN were noted to cause varying widespread endocytic defects including atypical localization of endosomal proteins, aberrant endosomal trafficking in both neuronal and non-neuronal tissue and impairments in JC polyomavirus infection – a clathrin-mediated endocytosis process (Dimitriadi et al., 2010). It was also documented that endocytic defects arise due to dysregulation of F-actin and calcium dynamics (Hosseinibarkooie et al., 2017). Plastin 3 (PLS3), an actin-bundling protein which interacts with F-actin and calcium, was shown to rescue motor neuron pathology, NMJ structure and function when overexpressed in a variety of SMA animal models (Oprea et al., 2008; Dimitriadi et al., 2010; Hosseinibarkooie et al., 2017). Similar rescue was observed when levels of neurocalcin delta, a negative regulator of endocytosis, were decreased (Riessland et al., 2017; Torres-Benito et al., 2019). Additionally, overexpression of coronin 1C, a calcium dependent F-actin binding protein which binds to PLS3, also rescued SMA defects in zebrafish (Hosseinibarkooie et al., 2017). Alternative roles of the SMN protein have been described in cell proliferation/differentiation (Grice and Liu, 2011), phosphatase and tensin homolog-mediated protein synthesis (Ning et al., 2010), energy homeostasis/mitochondrial function (Acsadi et al., 2009), priming of ribosomes to modulate translation (Lauria et al., 2020) and autophagy (Garcera et al., 2013; Custer and Androphy, 2014; Periyakaruppiah et al., 2016; Piras et al., 2017; Rodriguez-Muela et al., 2018; Sansa et al., 2021). Despite the various documented cellular roles of SMN, the specific interaction most pertinent to the development of SMA remains elusive. Treatment for SMA patients involves gene-targeted therapy in which the defective *SMN1* gene is being replaced or the *SMN2* gene splicing pattern is modulated.

### Therapeutic strategies

Three treatments are currently approved by the European Medicines Agency and the US Food and Drugs Administration: Nusinersen (Spinraza; Biogen), Onasemnogene abeparvovec (Zolgensma; Novartis) and Risdiplam (Evrysdi; Roche).

Nusinersen is the first gene therapy for SMA patients to be approved and is administered intrathecally. It costs up to $125,000 per dose and relies on the manipulation of *SMN2* gene splicing to increase the production of full-length, functional SMN protein. Exclusion of exon 7 is regulated by multiple surrounding elements; the most important one for nusinersen treatment is the intronic splicing silencer N1, deletion of which is known to significantly increase exon 7 inclusion in the *SMN2* mRNA transcript (Singh et al., 2006). Fundamentally, targeting this regulatory region via antisense oligonucleotides (ASOs) - modified chains of nucleotides with the ability to target a gene product of interest - forms the basis of nusinersen treatment. Effectiveness of this treatment was tested initially using a mild mouse model of SMA at embryonic, neonatal, and adult stages and it was noted that SMN protein levels were increased resulting in an improvement of the SMA phenotype (Hua et al., 2010). Additionally, the study by Hua et al. (2010) suggested that early treatment, at either the embryonic or neonatal stages resulted in stronger amelioration of the phenotypes observed in mild SMA mice. Furthermore, the ASO displayed an ability to significantly improve lifespan and motor function in severe mouse models of SMA (Passini et al., 2011). Subsequent clinical trials showed nusinersen to be most effective in infants treated before 6 months of life (Finkel et al., 2017) – however, more recent studies have reported improvements in patients of different stages and ages, covering the whole spectrum of SMA (Coratti et al., 2021; Łusakowska et al., 2023).

Onasemnogene abeparvovec is an *SMN1* gene replacement therapy which is administered as a single intravenous injection at a cost of $2,125,000 per dose. Expression of *SMN1* cDNA under the control of a ubiquitous promoter using intravenous delivery of the adeno-associated viral vector serotype 9 (AAV9) was efficient in transducing motor neurons in the spinal cord of mice (Dominguez et al., 2010). Through this technique, *SMN1* gene expression mediated by AAV9 significantly improved lifespan and motor function in SMA mouse models and non-human primates; similarly to nusinersen it was found that earlier intervention resulted in better outcomes (Valori et al., 2010; Meyer et al., 2015). In addition, clinical trials with type I SMA infants treated with onasemnogene abeparvovec displayed significant improvements in motor neuron function, although hepatotoxicity was observed as an adverse side effect (Mendell et al., 2017). Therefore, most patients require the corticosteroid prednisolone to mitigate the negative effects of liver toxicity (Chand et al., 2021). Overall, the aforementioned treatment is targeted at patients below 2 years of age, with bi-allelic mutations in the *SMN1* gene and up to three copies of *SMN2* (Wirth, 2021; Day et al., 2022; Ogbonmide et al., 2023).

Risdiplam, unlike its predecessors, is an orally administered small molecular drug that acts as an *SMN2* splice modulator with a cost of up to $340,000 per year. The molecule binds directly to *SMN2* pre-mRNA at two sites and promotes exon 7 inclusion by facilitating the recruitment of U1-snRNP1 particles to the splice donor site of intron 7, thus increasing the production of full-length SMN protein (Sivaramakrishnan et al., 2017). In preclinical trials, risdiplam was found to increase full-length functional SMN protein in both mild and severe SMA mice, drastically increasing lifespan and improving motor function defects (Poirier et al., 2018). Risdiplam treatment is offered to type I, II and III SMA patients aged 2 months and older with one to four copies of *SMN2* (Paik, 2022).

Other treatments in development include the myostatin inhibitor apitegromab. Myostatin is primarily expressed in skeletal muscle and serves to inhibit muscle growth (Sharma et al., 2015). Multiple preclinical SMA models have highlighted the effectiveness of myostatin inhibition in maintaining muscle mass and function (Rose et al., 2009; Feng et al., 2016). Apitegromab treatment has shown promise in milder type II and type III forms of disease (Barrett et al., 2021); the inhibitor has passed phase 1 and 2 trials having shown evidence to improve and/or stabilize motor function in SMA patients (Day et al., 2022). The identification of myostatin as a potential target for SMA treatment and the clinical benefit of apitegromab has led to the development of other therapies targeting myostatin as well as phase 3 trials of combination therapy with nusinersen or risdiplam (Day et al., 2022).

Fundamentally, while multiple pipeline treatments are in development and therapies are commercially available, there is no cure for SMA. It remains unclear which of the SMN function(s) result in disease pathogenesis. Firstly, it may be that the canonical role of SMN is the key cause of the disease, stemming from a global decrease in SMN protein function, subsequently interfering with the splicing of essential mRNA transcripts required for correct motor neuron development. There is also the possibility that one of the alternative functions of SMN, which is disrupted by SMN protein depletion results in a gradual deterioration of motor neurons. Our intention is to provide an overview of the role of autophagy in SMA which may in turn identify combinatory approaches that include SMN-dependent and -independent treatments for optimal benefits.

## Autophagy

**FIGURE 2:**
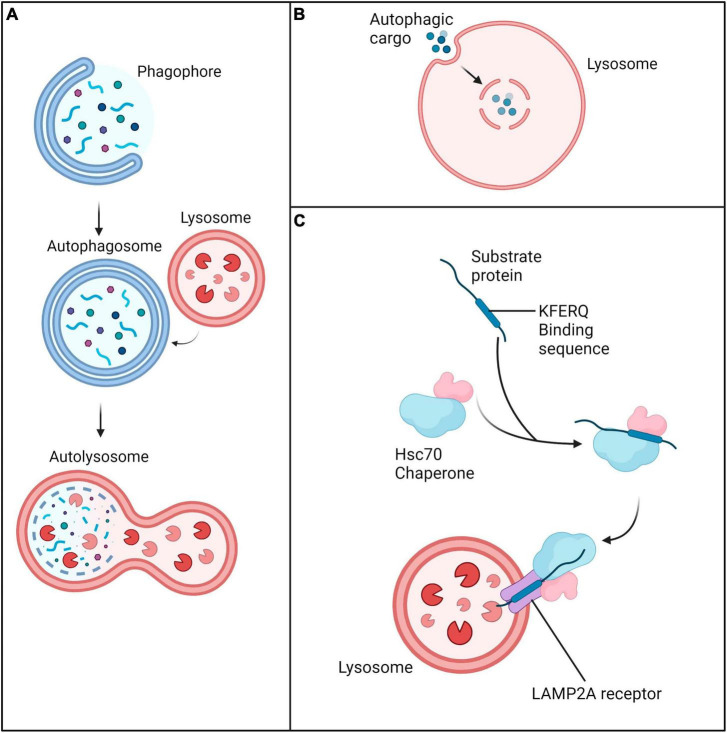
Three main forms of autophagy. **(A)** Macroautophagy is characterized by engulfment and transfer of cargo to lysosomes via an intermediary double membrane vesicle. **(B)** Microautophagy is characterized by direct invagination of cytoplasmic material into the lysosome. **(C)** Chaperone-mediated autophagy involves delivery of cargo marked by the Hsc70 chaperone complex to the lysosome via the LAMP2A receptor.

Autophagy is known to play a vital role in cell survival and maintenance of cellular integrity through proteostasis (Das et al., 2012). Autophagy can be upregulated in response to a wide range of stimuli; these include infection, starvation, oxidative stress, temperature, growth and development (Levine and Kroemer, 2008; Mizushima et al., 2008; Mizushima and Levine, 2010; Shang et al., 2011; Summers and Valentine, 2020). It is categorized into three types: macroautophagy, microautophagy and chaperone-mediated autophagy (Figure 2). While each type of autophagy ultimately results in lysosomal-mediated degradation of cytoplasmic components, the mode of delivery differs. Macroautophagy involves the formation of an intermediary double membrane vesicle, which engulfs cargo and transports it to the lysosome. Selective macroautophagy, in which specific cellular cargo are targeted for sequestration is dependent on the substrate; for example, the degradation of mitochondria (also known as mitophagy) is controlled by macroautophagy (Shimizu et al., 2014). In contrast, microautophagy involves direct invagination of cytoplasmic material into the lysosome. Chaperone-mediated autophagy is distinct from macro- and microautophagy due to the lack of transporter vesicles. During chaperone-mediated autophagy, cytoplasmic material containing the pentapeptide KFERQ motif are bound and chaperoned by the heat shock cognate 71 kDa protein Hsc70 which is recognized by the lysosomal LAMP2A receptor.

Macroautophagy (henceforth referred to as autophagy) is the main form of autophagy used for the clearance of aggregated proteins and damaged organelles due to its bulk degradation capabilities. As this review focuses on macroautophagy, microautophagy and chaperone-mediated autophagy will not be discussed; they have been extensively described in reviews by Li et al. (2012) and Kaushik and Cuervo (2018), respectively. During autophagy, the cytoplasmic material marked for degradation is sequestered by a nascent membrane known as the phagophore, which is believed to originate from the endoplasmic reticulum (Axe et al., 2008). The phagophore fuses with itself to form the autophagosome; upon completion of this process, the autophagosome fuses with a lysosome to form an autolysosome where lysosomal enzymes facilitate the degradation of cargo (Yorimitsu and Klionsky, 2005). Ultimately, autophagy occurs via five distinct stages: (i) autophagy induction, (ii) vesicle nucleation (iii) vesicle elongation (iv) docking and lysosomal fusion and (v) degradation. The ability of the pathway to progress through these stages is referred to as autophagic flux.

**FIGURE 3:**
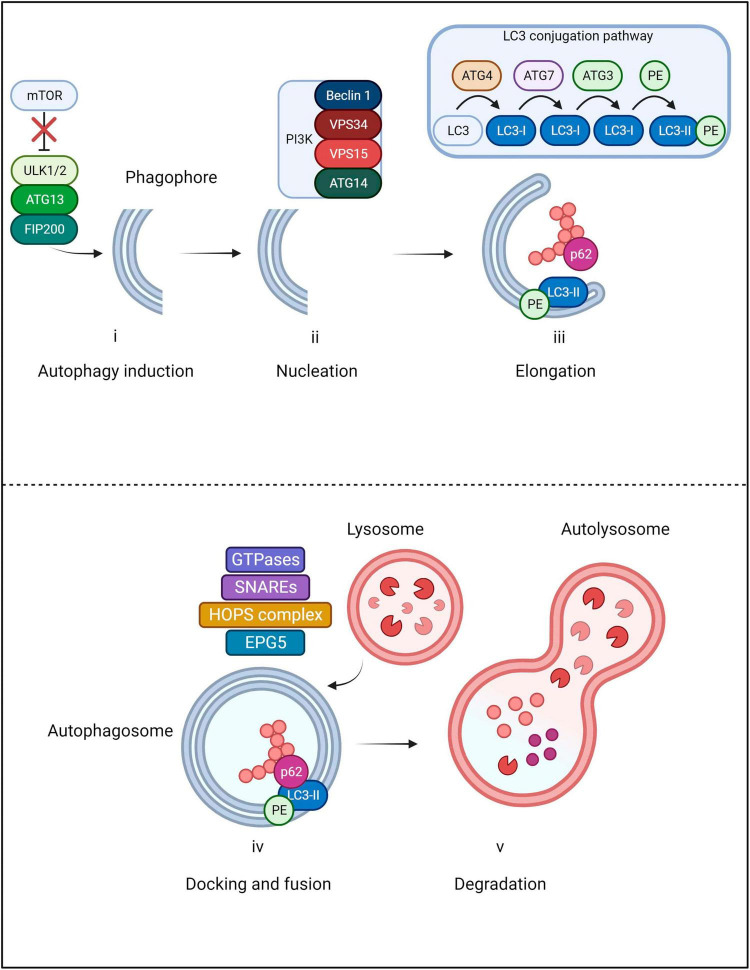
Main steps of macroautophagy. The pathway begins with the initiation step in which mTOR dissociates from the ULK1/2-ATG13-FIP200 autophagy induction complex in order for the phagophore to be formed. Subsequently, nucleation of the phagophore is orchestrated by a PI3K complex composed of Beclin 1, VPS34, VPS15 and ATG14. As the vesicle elongates, LC3 undergoes proteolytic cleavage by ATG4 into LC3-I. LC3-I is then bound by ATG7 and ATG3 and finally conjugated by phosphatidylethanolamine (PE); the resultant form is known as LC3-II. Once LC3-II incorporates into the autophagosome membrane, the extending ends join, engulfing cellular cargo. During the process, p62 delivers ubiquitinated cargo to the autophagosome where it binds with LC3-II. The autophagosome is then transported along microtubules to the lysosome which will dock and fuse through the action of small GTPases, SNAREs and the HOPS complex/EPG5 tethering factors. Cellular cargo then undergoes lysosomal degradation and dismantled components are recycled.

Autophagy is characterized by a multi-step process in which cytoplasmic contents are sequestered within double-membrane vesicles known as autophagosomes (Figure 3). In selective autophagy, recognition and delivery of ubiquitinated cargo to the autophagosome is orchestrated by the autophagic receptor Sequestosome 1 (p62/SQSTM1), hereafter referred to as p62 (Kumar et al., 2022). Initiation of the process begins with the dissociation of the mechanistic target of rapamycin (mTOR) from the ULK1/2-ATG13-FIP200 autophagy induction complex (Jung et al., 2009). Subsequent nucleation of the phagophore is orchestrated by a Class III phosphatidylinositol 3-kinase (PI3K) complex composed of Beclin 1, VPS34, VPS15 and ATG14 (Itakura et al., 2008; Burman and Ktistakis, 2010). The nucleated vesicle then expands through maturation of the membrane, guided by an ATG12-ATG5-ATG16L1 complex which catalyzes insertion of LC3 into the vesicle (Fujita et al., 2008). Prior to LC3 insertion into the membrane, LC3 undergoes proteolytic processing by ATG4 into LC3-I, subsequently bound by ATG7 and ATG3 and lastly, conjugated by phosphatidylethanolamine (PE) - the resulting mature form of LC3 is referred to as LC3-II (Kirisako et al., 2000; Ohsumi, 2001; Mizushima et al., 2011). Following LC3-II incorporation, the extending ends of the phagophore fuse with one another, forming the double membraned autophagosome which engulfs a segment of cytoplasm and cargo (Yorimitsu and Klionsky, 2005). The autophagosome is transported along microtubules and fuses with a lysosome to form an autolysosome; the cargo is then degraded by hydrolytic proteases (Pankiv and Johansen, 2010). The docking and fusion of the lysosome to the autophagosome is enabled through the action of small GTPases (such as RAB7), SNAREs and the HOPS complex/EPG5 tethering factors (Lörincz and Juhász, 2020). Ultimately, dismantled cellular components are actively transported for reconstitution into new macromolecules or alternatively, for energy metabolism (Yorimitsu and Klionsky, 2005).

Autophagy is upregulated to act as a protective process to promote survival (Kataura et al., 2022; Yang et al., 2023). However, impaired or excessive autophagy can be detrimental and could result in the accumulation of protein aggregates – a characteristic of many neurodegenerative disorders. The autophagic pathway is particularly known to be perturbed in neurodegenerative disorders such as Alzheimer’s disease, Parkinson’s disease, amyotrophic lateral sclerosis (ALS) (Ravikumar et al., 2004; Bandyopadhyay and Cuervo, 2007; Hetz et al., 2009; Sarkar et al., 2011; Nilsson and Saido, 2014) and is emerging as a popular topic of investigation in SMA pathogenesis.

## Autophagy dysregulation in SMA

### Autophagosomes

An increasing body of *in vitro* and *in vivo* evidence suggests dysregulation of autophagy as a key factor in SMA pathogenesis (Table 1). LC3-I conversion to LC3-II is considered a general marker of autophagosome formation, while autophagosome abundance is linked with LC3-II protein levels (Mizushima et al., 2004). The presence of autophagosomes and autolysosomes were analyzed in the neurites and cytoplasm of Smn-depleted motor neurons (Garcera et al., 2013). Motor neurons were transduced with lentivirus containing short hairpin RNA sequences targeting specific sites of mouse Smn and it was identified that Smn reduction resulted in an increased autophagosome and autolysosome number compared to controls (Garcera et al., 2013). This increase was observed in both motor neuron soma and neurites. Similar findings were also recapitulated *in vivo*; LC3-II levels were increased in more severe SMA mice [*Smn(-/-)*; *SMN2]* motor neurons, further reinforcing autophagy dysregulation in SMA (Garcera et al., 2013). A study by Custer and Androphy (2014) highlighted similar findings. An NSC-34 cell line – a hybrid line produced by fusing mouse neuroblastoma cells N18TG2 and motor neurons from mouse embryo spinal cords (Cashman et al., 1992), was transfected with GFP-LC3. The latter is known to appear as discrete puncta following lipidation and incorporation to the autophagosome membrane (Custer and Androphy, 2014). It was identified that Smn-depleted cultures displayed increased autophagic puncta, suggesting an increase in autophagosome number. The accumulation of LC3-positive puncta is not unique to the NSC-34 model; SMA-derived human fibroblasts transfected with LC3-GFP also displayed an increased number of puncta/cells, further highlighting an increase in autophagosome number in SMA patients. In agreement with these findings, Periyakaruppiah et al. (2016) also demonstrated elevated number of autophagosomes in severe SMA mice [*Smn(-/-); SMN2]* by detecting increased LC3-II levels in their spinal cord motor neurons. These findings were also echoed in the SMNdelta7 mouse model, presenting a severe SMA phenotype; lumbar spinal cord motor neurons obtained from SMNdelta7 mice displayed increased LC3-II and Beclin 1 – a protein component of the vesicle nucleation complex, indicating an increased number of autophagosomes (Piras et al., 2017).

**TABLE 1:** Summary of the autophagic perturbations reported in SMA.

SMA model	Autophagic marker	Autophagosome number	Autophagic flux	Findings	References
Cultured Smn-depleted mice motor neurons More severe SMA mice (*Smn-/-*; *SMN2)*	LC3-II Beclin 1	Increased	Unaffected	Treatment with the lysosomal proteolysis inhibitor bafilomycin A1 increases LC3-II, suggesting autophagic flux to be unaffected when Smn is reduced. Inhibition of the autophagic regulator Beclin 1 significantly delays neuronal degeneration.	Garcera et al., 2013
NSC-34 cell line Cultured SMA patient fibroblasts (Type I child line 3813T; heterozygous mother line 3814T) Taiwanese severe SMA mouse model (*Smn-/-; SMN2*^tg/0^*)*	LC3-II p62	Increased	Reduced	Smn depleted cultures transfected with GFP-LC3 display an increase in autophagosome numbers. Treatment with the autophagic inducer rapamycin was followed by assessment of p62 protein levels – accumulation of p62 was indicative of reduced autophagic flux.	Custer and Androphy, 2014
Cultured Smn-depleted mice motor neurons More severe SMA mice (*Smn-/-*; *SMN2)*	LC3-II p62	Increased	Reduced	p62 levels are significantly increased in spinal cord motor neurons of SMA mice, suggesting reduced autophagic flux. Treatment with the autophagic inhibitor bafilomycin A1 decreases Smn levels in control cells; treatment with the autophagy inducer rapamycin increases Smn levels in control cells.	Periyakaruppiah et al., 2016
Severe SMA mice (*Smn-/-*; *SMN2; SMN*Δ7)	LC3-II Beclin 1 p62	Increased	Unaffected	p62 levels remain unaltered in severe SMA mice suggesting autophagic flux not to be affected. Treatment with the autophagic inhibitor 3-methyladenine results in reduced autophagosome number, increased Smn levels and prolonged lifespan.	Piras et al., 2017
Severe SMA mice (*Smn-/-*; *SMN2; SMN*Δ7) SMA patient fibroblasts (Types I, II, III)	LC3-II p62	Increased	Reduced	Exposure to lysosomal inhibitors display a defective flux as evident by an increase in p62 protein levels, implying a reduction in autophagosome clearance. LC3-II levels are increased in the presence of lysosomal inhibitors; LC3 levels remain unchanged in SMA patients further suggesting increased levels of LC3-II are due to reduced autophagic flux rather than increased autophagosome formation. SMN protein is regulated by autophagy; i.e., starvation-induced autophagy causes decreased SMN levels – inhibition of autophagy increases SMN levels in starved cells. SMN protein interacts with p62; increased p62 levels resulted in further SMN protein reduction and elevated mTOR activity.	Rodriguez-Muela et al., 2018
SMA patient fibroblasts SMA patient muscle More severe SMA mice (*Smn-/-; SMN2)*	LC3-II p62 mTOR	Increased in motor neurons Decreased in fibroblasts Decreased in skeletal muscle	Reduced in motor neurons Reduced in fibroblasts Increased in skeletal muscles	LC3-II levels decrease in SMA patient muscle and fibroblasts but increase in motor neurons, suggesting tissue specific outcomes of SMN reduction. p62 decreases in SMA mice muscle suggesting an increase in autophagic flux. Conversely, p62 increases in SMA patient fibroblasts, suggesting a reduced autophagic flux. Similarly, p62 is increased in motor neurons, indicative of a reduced flux. mTOR signaling is found to be increased in motor neurons (autophagy inhibited); decreased in skeletal muscle and fibroblasts (autophagy activated).	Sansa et al., 2021

### Autophagic flux

Elevated numbers of autophagosomes could be the result of increased synthesis or reduced degradation, the latter being the result of impaired autophagic flux. To determine the cause of increased LC3-II levels, Smn-depleted motor neurons were treated with bafilomycin A1 – a lysosomal degradation inhibitor (Yamamoto et al., 1998; Garcera et al., 2013). LC3-II levels are expected to increase in the presence of bafilomycin A1 demonstrating an efficient autophagic flux (Menzies et al., 2012). Interestingly, bafilomycin-treated Smn motor neurons displayed a further increase in LC3-II compared to non-treated Smn controls (Garcera et al., 2013), an observation which would indicate some degree of functional autophagic flux. While the authors initially stated that autophagic flux was unaffected (Garcera et al., 2013), subsequent studies by the same group identified that flux was in fact reduced in Smn motor neurons by using a more comprehensive analysis (Periyakaruppiah et al., 2016; Sansa et al., 2021). It should be noted that even a partial blockage during the autophagy process could also result in LC3-II increase when bafilomycin A1 treatment is applied; this could be incorrectly interpreted as an unaffected flux (Klionsky et al., 2012). It is therefore important for researchers to assess LC3-II turnover in the presence of bafilomycin A1 with appropriate positive controls such as rapamycin treatment as well as examining the degradation of the autophagy specific substrate p62 (Klionsky et al., 2012).

To further clarify whether the accumulation of autophagosomes is due to an impaired autophagic flux or enhanced generation of autophagosomes, Custer and Androphy (2014) treated NSC-34 cultures with the autophagy activator rapamycin and expressed mRFP-LC3-GFP – a marker which allows for the differentiation between autophagosomes and autolysosomes by initially appearing yellow and subsequently transitioning to red after successful fusion with the lysosome (Custer and Androphy, 2014). An increased ratio of yellow puncta was observed in the Smn-depleted cultured cells treated with rapamycin, highlighting a failure of the autophagosome to fuse with the lysosome and thus, pointing to a reduced autophagic flux. Autophagic flux was further examined by assessing levels of the p62 protein, which is known to be degraded together with bound ubiquitinated substrates by autophagy following fusion with the lysosome. Increased p62 protein levels were observed in the NSC-34 cell line indicative of reduced autophagic flux (Custer and Androphy, 2014). Importantly, these findings were replicated in human SMA patient fibroblasts, as well as in the Taiwanese severe SMA mouse model (*Smn-/-; SMN2*^tg^*/^0^*) – animals carrying the Hung targeted deletion of murine Smn and a human *SMN2* transgene (Hsieh-Li et al., 2000; Custer and Androphy, 2014). However, Piras et al. (2017) found p62 levels to be unchanged in severe SMNdelta7 mice suggesting flux not to be affected. It is noteworthy that Custer and Androphy (2014) employed a more severe SMA mouse model, while Piras et al. (2017) drew conclusions from severe SMA mice which may be the cause of the discrepancies observed. Agreeing with the findings of Custer and Androphy (2014) and contrasting the findings of Garcera et al. (2013) and Piras et al. (2017), it was found that p62 protein levels were also significantly increased in the spinal cord motor neurons of more severe SMA mice, further indicating a reduced autophagic flux (Periyakaruppiah et al., 2016). Moreover, wild-type mouse motor neurons cultured with bafilomycin A1 in the presence of neurotrophic factors displayed increased LC3-II and reduced Smn levels relative to untreated controls, suggesting that inhibitors of autophagic flux resulted in reduced Smn protein levels in healthy controls (Periyakaruppiah et al., 2016). In line with these findings, SMA patient fibroblasts treated with lysosomal inhibitors displayed an accumulation of p62, while LC3-II increase was significantly lower than the increase observed in control fibroblasts, suggesting that autophagosome formation was not impaired (Rodriguez-Muela et al., 2018) A potential mechanism that could underlie the autophagic flux defects observed in SMA may be due to the disrupted SNARE complex assembly, an observation that has recently been described following SMN depletion (Kim et al., 2023). Fundamentally, the SNARE complex is required for autophagosome-lysosome fusion and therefore, when impaired, autophagic flux is expected to be reduced (Mohamud et al., 2018). SNAP25, a core component of the SNARE complex that is capable of mediating autophagosome-lysosome fusion was significantly reduced in more severe SMA mice (Mu et al., 2018; Kim et al., 2023). However, the interaction between SNAP25 and SMN has not been explored in the context of autophagy dysregulation. Overall, these studies point to an increase in autophagosome number, but it remains controversial whether autophagic flux is affected in SMA.

### Drug modulators of autophagy

Autophagic drug modulators have also been utilized to study the effect of autophagy in SMA. As mTOR is widely accepted as a major negative regulator of autophagy, rapamycin – an mTOR inhibitor, is commonly used to study autophagy activation (Noda and Ohsumi, 1998; Ravikumar et al., 2004; Berger et al., 2006). Wild-type mouse motor neurons cultured in the presence of neurotrophic factors and treated with rapamycin showed elevated Smn and LC3-II protein levels, while the same type of tissue when treated with bafilomycin A1 displayed reduced Smn protein levels (Periyakaruppiah et al., 2016). Conversely, administration of 3-methyladenine – which inhibits PI3K and subsequently autophagy initiation - reduced Beclin 1 and LC3-II and increased Smn protein levels in SMA cell cultures (Piras et al., 2017). Moreover, it was found that 3-methyladenine significantly increased the lifespan and number of motor neurons in SMA pups, suggesting that autophagy inhibition delayed motor neuron degeneration (Piras et al., 2017). While these studies seem contradictory, it should be noted that the findings of Periyakaruppiah et al. (2016) are based on control animals, whereas those by Piras et al. (2017) derive from severe SMA mice. Therefore, under normal conditions, activating autophagy is able to increase SMN levels. Moreover, it has been shown that rapamycin can increase autophagic flux in primary neurons (Rubinsztein and Nixon, 2010) and 3-methyladenine, surprisingly, presents a dual-role in autophagic regulation being able to inhibit the pathway but also promote autophagic flux due to its transient effects on varying classes of PI3K (Wu et al., 2010). The rescue observed by Piras et al. (2017) when administering 3-methyladenine may be a result of alleviating the defects in autophagic flux and the burden of autophagosome accumulation. Thus, the conclusions driven by the administration of these drugs may not be as straightforward as previously thought, and it is possible that the effect on autophagic flux is responsible for the contradictory findings.

### Autophagy-related regulators

Apart from studies on SMA focusing on (i) autophagosome number, (ii), autophagic flux and (iii) SMN endogenous levels following drug treatment, several studies have linked autophagy-related proteins to SMA. Diminished Smn protein levels are known to cause neurite degeneration and non-apoptotic cell death in spinal cord motor neurons of mice (Garcera et al., 2011). This was prevented by overexpression of Bcl-XL - a protein known to inhibit autophagy by binding to Beclin 1 (Pattingre et al., 2005) - without increasing Smn levels in embryonic mouse motor neurons (Garcera et al., 2011). Thus, Smn-depleted motor neurons expressing human Bcl-XL showed significant decreases in LC3-II protein levels (Garcera et al., 2013). This decrease was not observed in controls, suggesting the aforementioned Bcl-XL reduction of LC3-II levels to be specific to the dysregulation caused by Smn knockdown (Garcera et al., 2013).

Furthermore, calpain, a calcium-dependent protease involved in neuronal homeostasis has been suggested to play a role in Smn regulation (Cowan et al., 2008; Fuentes et al., 2010; Yamashima, 2013; de la Fuente et al., 2019). Elevated levels of free cytosolic calcium activate calpains, which in turn inhibit autophagy in an mTOR-independent manner (Kaiser et al., 2006; Williams et al., 2008; Gou-Fabregas et al., 2009). The reduction of endogenous calpain in SMA mice motor neurons resulted in increased autophagy and Smn protein levels (Periyakaruppiah et al., 2016).

Coimmunoprecipitation experiments in SMA mouse motor neurons revealed Smn to interact with p62 (Rodriguez-Muela et al., 2018). Smn protein depletion resulted in increased mTOR signaling activity that was characterized by (i) a reduction in autophagic activity and (ii) a decrease in lysosomal gene expression (Rodriguez-Muela et al., 2018). This was accompanied by increased p62 levels and subsequently a further Smn reduction, which in turn led to elevated mTOR activity (Rodriguez-Muela et al., 2018). In line with this cycle, autophagy induction by starvation in human fibroblasts gave rise to decreased SMN levels, whereas blocking autophagic activity with the lysosomal inhibitors ammonium chloride and leupeptin elevated SMN levels (Rodriguez-Muela et al., 2018). Furthermore, reducing p62 levels promoted motor neuron survival *in vitro* and increased the lifespan in the SMA fly model as well as SMNdelta7 severe SMA mice (Rodriguez-Muela et al., 2018). Ultimately, SMN depletion further exacerbated the observed autophagic dysregulation in SMA and overall, these findings suggest that SMN levels can be altered by targeting autophagy-related regulators.

### Tissue-specificity in SMA

It has been well established that SMA is not solely a motor neuron disease and that SMN depletion results in tissue specific aberrations and multi-organ dysfunction (Shababi et al., 2014; Sansa et al., 2021). In relation to autophagy and SMA, while the skeletal muscle of more severe SMA mice showed a decrease in autophagosome formation and an increase in autophagic flux, motor neurons displayed enhanced autophagosome formation and reduced autophagic flux (Sansa et al., 2021). Investigation into patient fibroblasts also revealed decreased LC3-II and increased p62 levels, suggesting decreased autophagosome number and autophagic flux, respectively. Additionally, it was noted that mTOR phosphorylation was reduced in patient fibroblasts and mouse skeletal muscle cells but increased in motor neurons (Sansa et al., 2021). Ultimately, these studies highlight a dysregulation of autophagy and an accumulation of autophagosomes as contributors to SMA pathogenesis. However, the effect of SMN depletion on autophagic flux remains contradictory; it is still poorly explored whether autophagy is in fact a destructive or beneficial factor underlying SMA development. Furthermore, these findings highlight the challenges that would be presented by targeting autophagy in SMA treatment given the observed differences in tissue-dependent autophagic activity.

## Autophagy: a key player in SMA onset with therapeutic implications

### Autophagosome accumulation: a key feature in SMA

The autophagic pathway is vital for homeostasis; cell functionality and survival are significantly reduced when autophagosome formation is compromised and autophagic bodies accumulate (Garcera et al., 2013; Custer and Androphy, 2014; Periyakaruppiah et al., 2016; Piras et al., 2017; Rodriguez-Muela et al., 2018; Sansa et al., 2021). In normal circumstances, neurons particularly rely on the autophagic pathway to maintain a healthy environment (Nixon, 2013). However, autophagy is considered a ‘double-edged sword’ which can both contribute to and protect from neuronal damage (Martinet et al., 2009). In neurodegenerative diseases where mutant protein aggregates are observed, it has been suggested that the autophagosome accumulation results in impaired intracellular trafficking and may also become the source of cytotoxic products (Ravikumar et al., 2004; Wong and Cuervo, 2010). While there are no recorded observations of aggregated proteins accumulating in SMA to date, autophagosome accumulation is known to perturb axonal transport which in turn results in neuronal degeneration; a hallmark of SMA pathogenesis observed *in vitro* and *in vivo* models (Rossoll et al., 2003; Burghes and Beattie, 2009; Garcera et al., 2011; Lee et al., 2011).

### Autophagy modulators: novel therapeutic targets

Pharmacological inhibition of autophagy using 3-methyladenine reduced autophagosome formation, delayed motor neuron degeneration and significantly improved lifespan in severe SMA mice (Piras et al., 2017). These findings imply that while the underlying cause of axonal deficiencies observed in SMA remains unclear, induction of autophagy and subsequent accumulation of autophagosomes may be a major contributing factor; a hypothesis supported by the finding that inhibition of Beclin 1, a regulator of autophagy required for induction, significantly delayed neuronal degeneration (Garcera et al., 2013). Moreover, autophagy induction with rapamycin resulted in decreased SMN and SMNΔ7 levels, suggesting both to be directly regulated by autophagy (Rodriguez-Muela et al., 2018). Furthermore, treatment with rapamycin demonstrated a failure in autophagosome-lysosome fusion and reduced lifespan in severe SMA mice (Custer and Androphy, 2014). While these findings demonstrate a possibility of therapy by inhibiting autophagy, the findings of Periyakaruppiah et al. (2016) put this scenario into question as autophagy induction has also been shown to increase full length SMN protein levels. Nevertheless, three studies agree that autophagic flux is reduced (Custer and Androphy, 2014; Periyakaruppiah et al., 2016; Rodriguez-Muela et al., 2018). Custer and Androphy (2014) suggested the use of new rapamycin analogues as these may be beneficial to stimulate autophagy beyond the capabilities of traditional rapamycin and in turn, overcome the altered autophagic flux levels.

### ALS and SMA: autophagy markers in two histopathologically similar motor neuron diseases

As previously mentioned, autophagy has been implicated in a range of neurodegenerative disorders. A prime example is ALS, which presents with aggregation of ubiquitinated proteins (such as SOD1, FUS, and TDP-43), while sharing histopathological findings with SMA (Blokhuis et al., 2013; Comley et al., 2016). In ALS, motor neuron survival is directly affected by an impairment in the autophagic pathway. More specifically, axonal transport defects are suggested to be responsible for the autophagosome accumulation observed in ALS mice (Cozzi and Ferrari, 2022). Autophagy is known to be dysregulated in ALS at different steps of the pathway, which results in autophagosome accumulation and a defective autophagic flux (Song et al., 2012; Lee et al., 2015; Nguyen et al., 2019). The aggregation of ALS-associated SOD1 – an antioxidant enzyme highly mutated in more than 20% of familial ALS cases (Corson et al., 1998; Gruzman et al., 2007; Berdyński et al., 2022) and TDP-43–a protein involved in RNA processing regulation (Hergesheimer et al., 2019) occurs in motor neuron axons and results in autophagy dysregulation (Sasaki, 2011; Wei, 2014; Budini et al., 2017). In ALS, autophagy inhibition by 3-methyladenine does not protect from motor neuron degeneration (as seen in SMA) and was suggested to induce TDP-43 aggregation (Caccamo et al., 2009). On the other hand, autophagy induction by rapamycin reduced TDP-43 accumulation, rescued mRNA processing (a key role of the SMN protein), resulted in migration of TDP-43 to its proper nuclear compartment (Caccamo et al., 2009) and significantly improved neuronal survival in ALS models (Barmada et al., 2014). In line with the protective role of autophagy in motor neuron diseases, it has been shown that motor neuron death due to glutamate-induced toxicity could be the result of autophagic pathway impairment (Fulceri et al., 2011). Therefore, the role of autophagy in ALS is especially important in the context of SMA given the similarities of the two diseases. The use of rapamycin showed consistency in its ability to improve disease symptoms in both ALS and SMA, while 3-methyladenine highlighted contrasting views (Caccamo et al., 2009; Periyakaruppiah et al., 2016). However, it must be noted that while SMA is a monogenic disease and primarily affects lower motor neurons, the genetic cause of ALS is complex affecting both upper and lower motor neurons (Burghes and Beattie, 2009; Ragagnin et al., 2019). Therefore, while the two diseases share histopathological similarities, the differences observed in modulating autophagy may be due to the distinct mechanisms of neuromuscular disruption underlying ALS but not SMA pathogenesis (Comley et al., 2016).

### Autophagy regulators: understanding the mechanisms behind autophagy dysregulation in SMA

Despite an established body of evidence that autophagosome number increases across a variety of SMA models (Garcera et al., 2013; Custer and Androphy, 2014; Periyakaruppiah et al., 2016; Piras et al., 2017; Rodriguez-Muela et al., 2018; Sansa et al., 2021), it is still debated whether autophagic flux is affected and subsequently whether autophagy itself is increased or decreased. Findings point to autophagy perturbations in SMA and an interaction between SMN and p62 has been suggested (Rodriguez-Muela et al., 2018). While it is documented that the SMN protein levels are regulated by the autophagic pathway, it may be of benefit to further understand the interactions between SMN and autophagy regulators. It was previously established that SMN depletion resulted in decreased levels of UBA1 (Lambert-Smith et al., 2020). Mutations in UBA1 are shown to induce a rare, non-SMN gene associated form of SMA with similar clinical symptoms (Ramser et al., 2008). UBA1 was able to regulate autophagy in an ATG7- and ATG3-independent manner in *Drosophila*, although the exact mechanism remains speculative (Chang et al., 2013). Thus, UBA1 decrease, which inevitably follows SMN depletion, may be contributing to impairments in the autophagic machinery. Moreover, various autophagy marker mRNAs (primarily the autophagy regulator Beclin 1, Atg5, LC3 and p62/SQSTM1) were shown to be elevated in severe SMA mouse models, suggesting dysregulated autophagy induction as a compensatory mechanism in response to disease progression (Oliván et al., 2016). Injection of tetanus toxic heavy chain (TTC) – a recombinant protein known to display neurotrophic capabilities – in SMA mice resulted in neuroprotective effects (Oliván et al., 2016). Interestingly, TTC administration also downregulated Beclin 1, Atg5, LC3 and p62 expression to wildtype levels which implied a return to constitutive autophagy function (Oliván et al., 2016) and highlighted autophagy regulators as potential therapeutic targets. Furthermore, Bcl-2 – a protein derived from the same family as the aforementioned Bcl-XL – is known to regulate autophagy by binding to Beclin 1 and inhibiting the pathway (Marquez and Xu, 2012). This is noteworthy as SMN has been shown to directly interact with Bcl-2 (Iwahashi et al., 1997), further demonstrating the importance of examining the functional interactions of SMN with key autophagy players.

Recent findings suggest an important role for microRNAs (miRNAs) - small RNAs tasked with regulating post-transcriptional gene expression in the pathogenesis of several motor neuron diseases including SMA (Magri et al., 2018). A handful of miRNAs implicated in SMA have also been shown to have a regulatory effect in the autophagic pathway. For example, miRNA-206, a miRNA involved in skeletal muscle that drives development and regenerative pathways at the neuromuscular junction, was upregulated in SMA and potentially delayed motor neuron degeneration, albeit not enough to rescue motor neuron integrity (Valsecchi et al., 2015, 2020). miRNA-206 overexpression in severe SMA mice resulted in improved motor neuron function and lifespan in mammalian models (Valsecchi et al., 2015, 2020). These findings prove to be highly relevant as evidence has suggested that miRNA-206 overexpression also resulted in increased autophagy in head and neck squamous cell carcinoma cells (Li et al., 2020). Similarly, it was found that miRNA-9, which promotes neuronal function/differentiation by activating autophagy was decreased upon SMN depletion (Yuva-Aydemir et al., 2011; Zhang et al., 2015; Magri et al., 2018). Furthermore, miRNA-183, which constitutes a role in axon outgrown (Wang et al., 2017), was found to be overexpression in more severe SMA mice (Kye et al., 2014). Intriguingly, miRNA-183 was established as an inhibitor of the autophagic pathway (Huangfu et al., 2016; Yuan et al., 2018). Kye et al. (2014) suggested that reduced SMN protein levels alter miRNA expression and distribution in neurons, whereas inhibition of miRNA-183 rescued Smn-knockdown axonal phenotypes in rat-derived neurons. Moreover, overexpression of miRNA-23a, a regulator of oligodendrocyte differentiation, increased the lifespan of severe SMA mice and inhibited autophagy (Lin et al., 2013; Si et al., 2018; Kaifer et al., 2019). Similarly, it was found that increased miRNA-146, a regulator of the inflammatory response, induced autophagy, while upregulation of this miRNA was observed in SMA mice (Cheng et al., 2013; Sison et al., 2017; Roy, 2021). Lastly, more severe SMA mice showed a marked reduction of miRNA-132 - which was known to promote dendritic growth and synaptic function - in the spinal cord (Catapano et al., 2016). In contrast, miRNA-132 levels were increased in more severe SMA mice skeletal muscles (Catapano et al., 2016). Interestingly, miRNA-132 overexpression was able to inhibit autophagy and its knockdown resulted in increased LC3 and reduced p62, indicative of autophagy induction (Ucar et al., 2012). Together, these findings clearly highlight the effect of SMN depletion on autophagy regulators. The aforementioned studies reveal the extent to which abnormal miRNA expression is related to disease. Future studies could employ the use of miRNA mimics and inhibitors which are capable of enhancing or rescuing the downregulation of targets, respectively. Additionally, these miRNA mimics and inhibitors should be observed for their effects on the autophagic pathway in the context of SMA. Interestingly. miRNA-155 inhibition which is known to alleviate autophagic flux defects in a pancreatitis mouse model (Wan et al., 2019) was also able to prolong survival in *SOD1* mutant mice (Koval et al., 2013; Seto et al., 2018; Gallant-Behm et al., 2019), highlighting that miRNAs could also serve as potential therapeutic targets for SMA.

### Mitophagy

A hallmark of neurodegenerative disorders including SMA is the observed structural and functional mitochondrial defects prior to symptom development (Miller et al., 2016); thus, mitophagy – mitochondrial degradation via the macroautophagy pathway - should also be considered. Deguise et al. (2016) observed autophagic vacuoles (autophagosomes and autolysosomes) containing mitochondria in severe SMA mice, suggesting mitophagy to be activated. Importantly, muscle tissue in severe SMA mice revealed an accumulation of dysfunctional mitochondria with reduced clearance as well as a downregulation of mitochondria and lysosomal expressed genes (Chemello et al., 2023). The same study highlighted that apart from reduced mitochondrial clearance, proteins that mark mitochondria for mitophagy (i.e., PINK and Parkin) as well as p62 were also elevated indicating a decline in autophagic flux. Furthermore, Chemello et al. (2023) noted a downregulation of genes involved in lysosomal biogenesis, which may underlie a defect in autophagosome-lysosome fusion. Additionally, type I, II and III SMA patient muscle tissue analysis revealed reduced mitochondrial DNA content (Berger et al., 2003; Ripolone et al., 2015), confirming mitochondrial defects in human samples.

**FIGURE 4:**
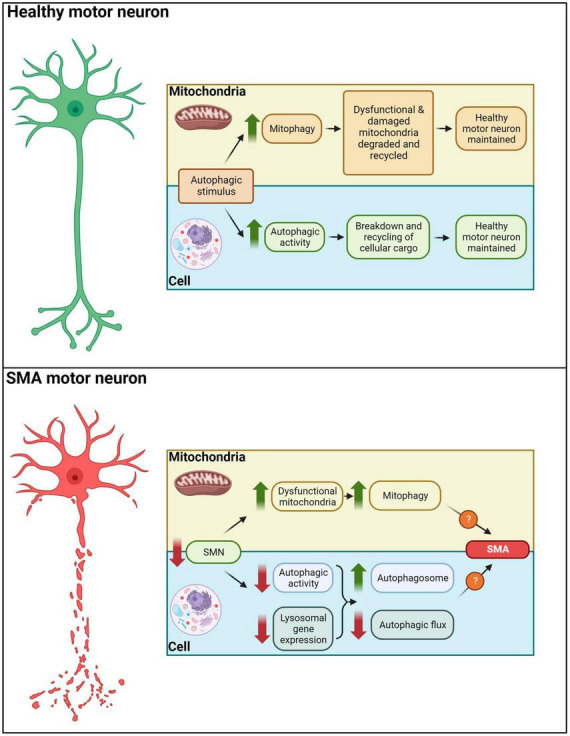
Autophagy in healthy and SMA motor neurons. In healthy motor neurons autophagic stimuli would lead to an increased mitophagy/autophagic activity, degradation and recycling of cellular cargo. In SMA motor neurons, decreased SMN levels result in dysfunctional mitochondria, reduced autophagic activity and downregulation of lysosomal gene expression. These aberrations drive autophagosome accumulation and diminished autophagic flux, potentially leading to apoptotic and non-apoptotic cell death in motor neurons of SMA patients.

Mitochondria play a key role in the activation of apoptosis (Xiong et al., 2014) and it has been shown that accumulation of autophagosomes resulted in the activation of the apoptotic pathway and subsequently, cell death (Mariño et al., 2014). Piras et al. (2017) hypothesized that the increase in autophagosomes and autolysosomes may be responsible for the degeneration of lower motor neurons in SMNdelta7 severe mice as the increase in autophagosomes is likely to result in increased apoptotic cell death. Moreover, an increase in apoptotic nuclei and reduced levels of antiapoptotic proteins have been observed in more severe SMA mice and patient’s motor neurons, while inhibition of apoptosis has been shown to block motor neuron cell death in SMA stem cell models (Sareen et al., 2012; Sansa et al., 2021). The observed mitochondrial damage is correlated with the severity of the disease; more severe SMA mice present with lower numbers of active mitochondria (Torres-Benito et al., 2011), suggesting that autophagy may in part influence disease phenotype. Therefore, it is possible that the presence of defective mitochondria results in autophagy activation which presents with reduced flux due to the downregulation of lysosomal genes. Thus, autophagosomes accumulate and subsequently signal the apoptotic pathway, resulting in motor neuron death. Ultimately, this may suggest that impaired autophagic flux – and not increased autophagy - could be the causative agent underlying autophagy-related development of symptoms in SMA (Figure 4).

The information presented herein highlights the autophagy networks perturbed in SMA and identifies these as a gateway to explore potential therapeutic interventions. While the potential of autophagy modulation holds significant promise, it is necessary to navigate through the appropriate considerations to overcome the boundaries of clinical applicability.

## Autophagy as a therapeutic target for SMA: considerations and limitations

It is paramount for SMA researchers to initially consider the stage of autophagy that is mainly affected in SMA. Given the contradictory findings regarding (a) autophagic flux and (b) the lack of verdict on whether autophagy is in fact upregulated in SMA, future research should be aimed at elucidating these discrepancies.

It would be beneficial to study fluorescent probes (i.e., mRFP-EGFP-LC3B) that are degraded by the autolysosome and show specificity for autophagic flux (Chapin et al., 2015). Moreover, the tissue-specificity of autophagic dysregulation in SMA could present a barrier in translating research evidence into clinical practice for SMA patients. Particularly, SMA patient-derived motor neurons displayed a reduced flux while skeletal muscle from SMA mice showed the opposite phenotype, and as such, the development of autophagy-targeted therapy for SMA must account for the varying levels of autophagic activity in different tissues (Sansa et al., 2021). This is equally as relevant when considering the different models used in these studies. For example, severe SMA mice indicated no change in flux according to Piras et al. (2017), although Rodriguez-Muela et al. (2018) documented reduced flux in the same model. On the other hand, more severe SMA mice and type I-III patient fibroblasts exhibited an impaired one (Sansa et al., 2021). Together, this suggests that further analysis is needed to elucidate the autophagic flux defects in severe SMA mice.

It would be of benefit to identify whether the observed therapeutic effects in the context of SMA are a result of autophagic inhibition or a rescue of flux defects in order to consider the stage of autophagy the intended therapy should be acting on. This is of particular importance when considering the literature findings of using the autophagic inhibitor 3-methyladenine (Piras et al., 2017), which we previously mentioned can play a dual role in modulating autophagy (Wu et al., 2010).

Besides, many of the known autophagic activators like rapamycin are known to act through mTOR inhibition; mTOR is also involved in autophagy independent pathways, such as lipid and nucleotide synthesis, protein synthesis, cell growth and immunosuppression (Saxton and Sabatini, 2017). For example, SMA patients present with a higher susceptibility to infection (Deguise and Kothary, 2017) and given the immunosuppression which follows prolonged mTOR inhibition, it may be more advantageous to consider compounds capable of activating autophagy independent of mTOR inhibition. Alternatively, clinicians would be required to regularly monitor liver and renal functions as well as cholesterol and triglycerides, a commonplace practice for patients taking rapamycin for the treatment of other diseases (Kahan et al., 2000).

When identifying potential new agents for treating SMA, one important consideration is that they should be capable of penetrating the blood-brain barrier. Furthermore, sex dependent limitations of treatments for SMA patients must be considered. This is specifically relevant in SMA where the protective capabilities of genetic modifiers such as PLS3 are known to be sex specific (Yanyan et al., 2014). For example, rapamycin-mediated increase in lifespan has been shown to be higher in female than in male mice across various doses (Miller et al., 2014). Therefore, future research should aim at highlighting any potential sex-specific differences following administration of autophagy modulators.

Finally, the identification of any autophagy-targeted treatment(s) should be considered complimentary to the current therapeutic strategies for SMA and administered at the earliest possible stage of the disease. As such, any potential treatments should be studied in combination with current SMA therapies to identify possible interactions.

## Conclusions

We have summarized the growing body of evidence which underlies the dysregulation of autophagy in SMA, characterized mainly by an increase in autophagosome number. We speculate that it is the late, degradative stage of autophagy which seems impaired in SMA due to a failure in the lysosome-autophagosome docking and fusion step. It is important to consider the stage at which autophagy becomes dysfunctional in SMA patients before autophagy-targeted treatments can be considered. Subsequently, addressing the outlined limitations of autophagy-targeted treatments would be pivotal in designing specific and accurate treatments for SMA patients. Future research directions should aim to identify (a) the effect of SMN depletion on autophagic flux and (b) the relationship between SMN and the most important autophagy regulators known to be impacted in SMA. We advocate for a redirected research focus in order to unravel the discrepancies regarding autophagic flux which may in turn identify novel modifiers of the disease and lead to more effective therapeutic strategies in SMA.

## Author contributions

SR: Writing—original draft, Writing—review and editing. MD: Writing—review and editing.

## References

[B1] Acsadi G. Lee I. Li X. Khaidakov M. Pecinova A. Parker G. (2009). Mitochondrial dysfunction in a neural cell model of spinal muscular atrophy. *J. Neurosci. Res.* 87 2748–2756. 10.1002/jnr.22106 19437551

[B2] Axe E. Walker S. Manifava M. Chandra P. Roderick H. Habermann A. (2008). Autophagosome formation from membrane compartments enriched in phosphatidylinositol 3-phosphate and dynamically connected to the endoplasmic reticulum. *J. Cell Biol.* 182 685–701. 10.1083/jcb.200803137 18725538 PMC2518708

[B3] Bandyopadhyay U. Cuervo A. (2007). Chaperone-mediated autophagy in aging and neurodegeneration: lessons from alpha-synuclein. *Exp. Gerontol.* 42 120–128. 10.1016/j.exger.2006.05.019 16860504

[B4] Barmada S. Serio A. Arjun A. Bilican B. Daub A. Ando D. (2014). Autophagy induction enhances TDP43 turnover and survival in neuronal ALS models. *Nat. Chem. Biol.* 10 677–685. 10.1038/nchembio.1563 24974230 PMC4106236

[B5] Barrett D. Bilic S. Chyung Y. Cote S. Iarrobino R. Kacena K. (2021). A randomized phase 1 safety, pharmacokinetic and pharmacodynamic study of the novel myostatin inhibitor apitegromab (SRK-015): a potential treatment for spinal muscular atrophy. *Adv. Ther.* 38 3203–3222. 10.1007/s12325-021-01757-z 33963971 PMC8189951

[B6] Béchade C. Rostaing P. Cisterni C. Kalisch R. La Bella V. Pettmann B. (1999). Subcellular distribution of survival motor neuron (SMN) protein: possible involvement in nucleocytoplasmic and dendritic transport. *Eur. J. Neurosci.* 11 293–304. 10.1046/j.1460-9568.1999.00428.x 9987032

[B7] Berdyński M. Miszta P. Safranow K. Andersen P. Morita M. Filipek S. (2022). SOD1 mutations associated with amyotrophic lateral sclerosis analysis of variant severity. *Sci. Rep.* 12:103. 10.1038/s41598-021-03891-8 34996976 PMC8742055

[B8] Berger A. Mayr J. Meierhofer D. Fötschl U. Bittner R. Budka H. (2003). Severe depletion of mitochondrial DNA in spinal muscular atrophy. *Acta Neuropathol.* 105 245–251. 10.1007/s00401-002-0638-1 12557011

[B9] Berger Z. Ravikumar B. Menzies F. Oroz L. Underwood B. Pangalos M. (2006). Rapamycin alleviates toxicity of different aggregate-prone proteins. *Hum. Mol. Genet.* 15 433–442. 10.1093/hmg/ddi458 16368705

[B10] Blokhuis A. Groen E. Koppers M. van den Berg L. Pasterkamp R. (2013). Protein aggregation in amyotrophic lateral sclerosis. *Acta Neuropathol.* 125 777–794. 10.1007/s00401-013-1125-6 23673820 PMC3661910

[B11] Bowerman M. Beauvais A. Anderson C. Kothary R. (2010). Rho-kinase inactivation prolongs survival of an intermediate SMA mouse model. *Hum. Mol. Genet.* 19 1468–1478. 10.1093/hmg/ddq021 20097679

[B12] Bowerman M. Murray L. Boyer J. Anderson C. Kothary R. (2012). Fasudil improves survival and promotes skeletal muscle development in a mouse model of spinal muscular atrophy. *BMC Med.* 10:24. 10.1186/1741-7015-10-24 22397316 PMC3310724

[B13] Budini M. Buratti E. Morselli E. Criollo A. (2017). Autophagy and its impact on neurodegenerative diseases: new roles for TDP-43 and C9orf72. *Front. Mol. Neurosci.* 10:170. 10.3389/fnmol.2017.00170 28611593 PMC5447761

[B14] Burghes A. Beattie C. (2009). Spinal muscular atrophy: why do low levels of survival motor neuron protein make motor neurons sick? *Nat. Rev. Neurosci.* 10 597–609. 10.1038/nrn2670 19584893 PMC2853768

[B15] Burman C. Ktistakis N. (2010). Autophagosome formation in mammalian cells. *Semin. Immunopathol.* 32 397–413. 10.1007/s00281-010-0222-z 20740284

[B16] Caccamo A. Majumder S. Deng J. Bai Y. Thornton F. Oddo S. (2009). Rapamycin rescues TDP-43 mislocalization and the associated low molecular mass neurofilament instability. *J. Biol Chem.* 284 27416–27424. 10.1074/jbc.M109.031278 19651785 PMC2785671

[B17] Cartegni L. Krainer A. (2002). Disruption of an SF2/ASF-dependent exonic splicing enhancer in SMN2 causes spinal muscular atrophy in the absence of SMN1. *Nat. Genet.* 30 377–384. 10.1038/ng854 11925564

[B18] Cashman N. Durham H. Blusztajn J. Oda K. Tabira T. Shaw I. (1992). Neuroblastoma x spinal cord (NSC) hybrid cell lines resemble developing motor neurons. *Dev. Dyn.* 194 209–221. 10.1002/aja.1001940306 1467557

[B19] Catapano F. Zaharieva I. Scoto M. Marrosu E. Morgan J. Muntoni F. (2016). Altered levels of MicroRNA-9, -206, and -132 in spinal muscular atrophy and their response to antisense oligonucleotide therapy. *Mol. Ther. Nucleic Acids* 5 e331. 10.1038/mtna.2016.47 27377135 PMC5014531

[B20] Chand D. Mohr F. McMillan H. Tukov F. Montgomery K. Kleyn A. (2021). Hepatotoxicity following administration of onasemnogene abeparvovec (AVXS-101) for the treatment of spinal muscular atrophy. *J. Hepatol.* 74 560–566. 10.1016/j.jhep.2020.11.001 33186633

[B21] Chang T. Shravage B. Hayes S. Powers C. Simin R. Wade Harper J. (2013). Uba1 functions in Atg7- and Atg3-independent autophagy. *Nat. Cell Biol.* 15 1067–1078. 10.1038/ncb2804 23873149 PMC3762904

[B22] Chapin H. C. Okada M. Merz A. Miller D. (2015). Quantifying autophagy’s magnitude in normal. *Aging* 7 419–434. 10.18632/aging.100765 26142908 PMC4505168

[B23] Chemello F. Pozzobon M. Tsansizi L. Varanita T. Quintana-Cabrera R. Bonesso D. (2023). Dysfunctional mitochondria accumulate in a skeletal muscle knockout model of Smn1, the causal gene of spinal muscular atrophy. *Cell Death Dis.* 14:162. 10.1038/s41419-023-05573-x 36849544 PMC9971247

[B24] Cheng H. Sivachandran N. Lau A. Boudreau E. Zhao J. Baltimore D. (2013). MicroRNA-146 represses endothelial activation by inhibiting pro-inflammatory pathways. *EMBO Mol. Med.* 5 1017–1034. 10.1002/emmm.201202318 23733368 PMC3721471

[B25] Comley L. Nijssen J. Frost-Nylen J. Hedlund E. (2016). Cross-disease comparison of amyotrophic lateral sclerosis and spinal muscular atrophy reveals conservation of selective vulnerability but differential neuromuscular junction pathology. *J. Comp. Neurol.* 524 1424–1442. 10.1002/cne.23917 26502195 PMC5063101

[B26] Coratti G. Cutrona C. Pera M. Bovis F. Ponzano M. Chieppa F. (2021). Motor function in type 2 and 3 SMA patients treated with Nusinersen: a critical review and meta-analysis. *Orphanet. J. Rare Dis.* 16:430. 10.1186/s13023-021-02065-z 34645478 PMC8515709

[B27] Corson L. Strain J. Culotta V. Cleveland D. (1998). Chaperone-facilitated copper binding is a property common to several classes of familial amyotrophic lateral sclerosis-linked superoxide dismutase mutants. *Proc. Natl. Acad. Sci. U. S. A.* 95 6361–6366. 10.1073/pnas.95.11.6361 9600970 PMC27707

[B28] Cowan C. Fan M. Fan J. Shehadeh J. Zhang L. Graham R. (2008). Polyglutamine-modulated striatal calpain activity in YAC transgenic huntington disease mouse model: impact on NMDA receptor function and toxicity. *J. Neurosci.* 28 12725–12735. 10.1523/JNEUROSCI.4619-08.2008 19036965 PMC6671821

[B29] Cozzi M. Ferrari V. (2022). Autophagy dysfunction in ALS: From transport to protein degradation. *J. Mol. Neurosci.* 72, 1456–1481. 10.1007/s12031-022-02029-3 35708843 PMC9293831

[B30] Crawford T. Pardo C. (1996). The neurobiology of childhood spinal muscular atrophy. *Neurobiol. Dis.* 3 97–110. 10.1006/nbdi.1996.0010 9173917

[B31] Custer S. Androphy E. (2014). Autophagy dysregulation in cell culture and animals models of spinal muscular atrophy. *Mol. Cell Neurosci.* 61 133–140. 10.1016/j.mcn.2014.06.006 24983518 PMC4135029

[B32] Das G. Shravage B. Baehrecke E. (2012). Regulation and function of autophagy during cell survival and cell death. *Cold Spring Harb. Perspect. Biol.* 4 a008813. 10.1101/cshperspect.a008813 22661635 PMC3367545

[B33] Day J. Howell K. Place A. Long K. Rossello J. Kertesz N. (2022). Advances and limitations for the treatment of spinal muscular atrophy. *BMC Pediatr.* 22:632. 10.1186/s12887-022-03671-x 36329412 PMC9632131

[B34] de la Fuente S. Sansa A. Periyakaruppiah A. Garcera A. Soler R. (2019). Calpain inhibition increases SMN protein in spinal cord motoneurons and ameliorates the spinal muscular atrophy phenotype in mice. *Mol. Neurobiol.* 56 4414–4427. 10.1007/s12035-018-1379-z 30327977 PMC6505520

[B35] Deguise M. Boyer J. McFall E. Yazdani A. De Repentigny Y. Kothary R. (2016). Differential induction of muscle atrophy pathways in two mouse models of spinal muscular atrophy. *Sci. Rep.* 6:28846. 10.1038/srep28846 27349908 PMC4924104

[B36] Deguise M. Kothary R. (2017). New insights into SMA pathogenesis: immune dysfunction and neuroinflammation. *Ann. Clin. Transl. Neurol.* 4 522–530. 10.1002/acn3.423 28695153 PMC5497530

[B37] Dimitriadi M. Sleigh J. N. Walker A. Chang H. C. Sen A. Kalloo G. (2010). Conserved genes act as modifiers of invertebrate SMN loss of function defects. *PLoS Genetics* 6:e1001172. 10.1371/journal.pgen.1001172 21124729 PMC2965752

[B38] Dominguez E. Marais T. Chatauret N. Benkhelifa-Ziyyat S. Duque S. Ravassard P. (2010). Intravenous SCAAV9 delivery of a codon-optimized SMN1 sequence rescues SMA mice. *Hum. Mol. Genet.* 20, 681–693. 10.1093/hmg/ddq514 21118896

[B39] Edens B. Ajroud-Driss S. Ma L. Ma Y. (2015). Molecular mechanisms and animal models of spinal muscular atrophy. *Biochim. Biophys. Acta* 1852 685–692. 10.1016/j.bbadis.2014.07.024 25088406 PMC12184990

[B40] Fallini C. Bassell G. Rossoll W. (2012). Spinal muscular atrophy: the role of SMN in axonal mRNA regulation. *Brain Res.* 1462 81–92. 10.1016/j.brainres.2012.01.044 22330725 PMC3360984

[B41] Farrar M. Kiernan M. (2015). The genetics of spinal muscular atrophy: progress and challenges. *Neurotherapeutics* 12 290–302. 10.1007/s13311-014-0314-x 25413156 PMC4404441

[B42] Feng Z. Ling K. Zhao X. Zhou C. Karp G. Welch E. (2016). Pharmacologically induced mouse model of adult spinal muscular atrophy to evaluate effectiveness of therapeutics after disease onset. *Hum. Mol. Genet.* 25 964–975. 10.1093/hmg/ddv629 26758873

[B43] Finkel R. McDermott M. Kaufmann P. Darras B. Chung W. Sproule D. (2014). Observational study of spinal muscular atrophy type I and implications for clinical trials. *Neurology* 83 810–817. 10.1212/WNL.0000000000000741 25080519 PMC4155049

[B44] Finkel R. Mercuri E. Darras B. Connolly A. Kuntz N. Kirschner J. (2017). Nusinersen versus sham control in infantile-onset spinal muscular atrophy. *N. Engl. J. Med.* 377 1723–1732. 10.1056/NEJMoa1702752 29091570

[B45] Fischer U. Liu Q. Dreyfuss G. (1997). The SMN-SIP1 complex has an essential role in spliceosomal snRNP biogenesis. *Cell* 90 1023–1029. 10.1016/s0092-8674(00)80368-2 9323130

[B46] Franck A. Lainé J. Moulay G. Lemerle E. Trichet M. Gentil C. (2019). Clathrin plaques and associated actin anchor intermediate filaments in skeletal muscle. *Mol. Biol. Cell* 30 579–590. 10.1091/mbc.E18-11-0718 30601711 PMC6589689

[B47] Fuentes J. Strayer M. Matera A. (2010). Molecular determinants of survival motor neuron (SMN) protein cleavage by the calcium-activated protease, calpain. *PLoS One* 5:e15769. 10.1371/journal.pone.0015769 21209906 PMC3012718

[B48] Fujita N. Itoh T. Omori H. Fukuda M. Noda T. Yoshimori T. (2008). The Atg16L complex specifies the site of LC3 lipidation for membrane biogenesis in autophagy. *Mol. Biol. Cell* 19 2092–2100. 10.1091/mbc.e07-12-1257 18321988 PMC2366860

[B49] Fulceri F. Ferrucci M. Lazzeri G. Paparelli S. Bartalucci A. Tamburini I. (2011). Autophagy activation in glutamate-induced motor neuron loss. *Arch. Ital. Biol.* 149 101–111. 10.4449/aib.v149i1.1259 21412719

[B50] Gallant-Behm C. Piper J. Lynch J. Seto A. Hong S. Mustoe T. (2019). A MicroRNA-29 Mimic (Remlarsen) Represses Extracellular Matrix Expression and Fibroplasia in the Skin. *J. Invest. Dermatol.* 139 1073–1081. 10.1016/j.jid.2018.11.007 30472058

[B51] Garcera A. Bahi N. Periyakaruppiah A. Arumugam S. Soler R. (2013). Survival motor neuron protein reduction deregulates autophagy in spinal cord motoneurons in vitro. *Cell Death Dis.* 4 e686. 10.1038/cddis.2013.209 PMC3702296 23788043

[B52] Garcera A. Mincheva S. Gou-Fabregas M. Caraballo-Miralles V. Lladó J. Comella J. (2011). A new model to study spinal muscular atrophy: neurite degeneration and cell death is counteracted by BCL-X(L) Overexpression in motoneurons. *Neurobiol. Dis.* 42 415–426. 10.1016/j.nbd.2011.02.003 21333739

[B53] Giesemann T. Rathke-Hartlieb S. Rothkegel M. Bartsch J. Buchmeier S. Jockusch B. (1999). A role for polyproline motifs in the spinal muscular atrophy protein SMN. Profilins bind to and colocalize with smn in nuclear gems. *J. Biol. Chem.* 274 37908–37914. 10.1074/jbc.274.53.37908 10608857

[B54] Gou-Fabregas M. Garcera A. Mincheva S. Perez-Garcia M. Comella J. Soler R. (2009). Specific vulnerability of mouse spinal cord motoneurons to membrane depolarization. *J. Neurochem.* 110 1842–1854. 10.1111/j.1471-4159.2009.06278.x 19627436

[B55] Grice S. Liu J. (2011). Survival motor neuron protein regulates stem cell division, proliferation, and differentiation in Drosophila. *PLoS Genet.* 7:e1002030. 10.1371/journal.pgen.1002030 21490958 PMC3072375

[B56] Gruzman A. Wood W. Alpert E. Prasad M. Miller R. Rothstein J. (2007). Common molecular signature in SOD1 for both sporadic and familial amyotrophic lateral sclerosis. *Proc. Natl. Acad. Sci. U. S. A.* 104 12524–12529. 10.1073/pnas.0705044104 17636119 PMC1941502

[B57] Hergesheimer R. Chami A. de Assis D. Vourc’h P. Andres C. Corcia P. (2019). The debated toxic role of aggregated TDP-43 in amyotrophic lateral sclerosis: a resolution in sight? *Brain* 142 1176–1194. 10.1093/brain/awz078 30938443 PMC6487324

[B58] Hetz C. Thielen P. Matus S. Nassif M. Court F. Kiffin R. (2009). XBP-1 deficiency in the nervous system protects against amyotrophic lateral sclerosis by increasing autophagy. *Genes Dev.* 23 2294–2306. 10.1101/gad.1830709 19762508 PMC2758741

[B59] Hosseinibarkooie S. Schneider S. Wirth B. (2017). Advances in understanding the role of disease-associated proteins in spinal muscular atrophy. *Expert. Rev. Proteomics* 14 581–592. 10.1080/14789450.2017.1345631 28635376

[B60] Hsieh-Li H. Chang J. Jong Y. Wu M. Wang N. Tsai C. (2000). A mouse model for spinal muscular atrophy. *Nat. Genet.* 24 66–70. 10.1038/71709 10615130

[B61] Hua Y. Sahashi K. Hung G. Rigo F. Passini M. Bennett C. (2010). Antisense correction of SMN2 splicing in the CNS rescues necrosis in a type III SMA mouse model. *Genes Dev.* 24 1634–1644. 10.1101/gad.1941310 20624852 PMC2912561

[B62] Huangfu L. Liang H. Wang G. Su X. Li L. Du Z. (2016). miR-183 regulates autophagy and apoptosis in colorectal cancer through targeting of UVRAG. *Oncotarget* 7 4735–4745. 10.18632/oncotarget.6732 26717041 PMC4826239

[B63] Itakura E. Kishi C. Inoue K. Mizushima N. (2008). Beclin 1 forms two distinct phosphatidylinositol 3-kinase complexes with mammalian Atg14 and UVRAG. *Mol. Biol. Cell* 19 5360–5372. 10.1091/mbc.e08-01-0080 18843052 PMC2592660

[B64] Iwahashi H. Eguchi Y. Yasuhara N. Hanafusa T. Matsuzawa Y. Tsujimoto Y. (1997). Synergistic anti-apoptotic activity between Bcl-2 and SMN implicated in spinal muscular atrophy. *Nature* 390 413–417. 10.1038/37144 9389483

[B65] Jung C. Jun C. Ro S. Kim Y. Otto N. Cao J. (2009). ULK-Atg13-FIP200 complexes mediate mTOR signaling to the autophagy machinery. *Mol. Biol. Cell* 20 1992–2003. 10.1091/mbc.e08-12-1249 19225151 PMC2663920

[B66] Kahan B. Napoli K. Kelly P. Podbielski J. Hussein I. Urbauer D. (2000). Therapeutic drug monitoring of sirolimus: correlations with efficacy and toxicity. *Clin. Transplant.* 14 97–109. 10.1034/j.1399-0012.2000.140201.x 10770413

[B67] Kaifer K. Villalón E. O’Brien B. Sison S. Smith C. Simon M. (2019). AAV9-mediated delivery of miR-23a reduces disease severity in Smn2B/-SMA model mice. *Hum. Mol. Genet.* 28 3199–3210. 10.1093/hmg/ddz142 31211843 PMC6859438

[B68] Kaiser M. Maletzki I. Hülsmann S. Holtmann B. Schulz-Schaeffer W. Kirchhoff F. (2006). Progressive loss of a glial potassium channel (KCNJ10) in the spinal cord of the SOD1 (G93A) transgenic mouse model of amyotrophic lateral sclerosis. *J. Neurochem.* 99 900–912. 10.1111/j.1471-4159.2006.04131.x 16925593

[B69] Kashima T. Manley J. L. (2003). A negative element in SMN2 exon 7 inhibits splicing in spinal muscular atrophy. *Nat. Genet.* 34 460–463. 10.1038/ng1207 12833158

[B70] Kataura T. Sedlackova L. Otten E. Kumari R. Shapira D. Scialo F. (2022). Autophagy promotes cell survival by maintaining NAD levels. *Dev. Cell* 57 2584–2598. 36413951 10.1016/j.devcel.2022.10.008 PMC11475545

[B71] Kaushik S. Cuervo A. (2018). The coming of age of chaperone-mediated autophagy. *Nat. Rev Mol. Cell Biol.* 19 365–381. 10.1038/s41580-018-0001-6 29626215 PMC6399518

[B72] Kim J. Jha N. Awano T. Caine C. Gollapalli K. Welby E. (2023). A spinal muscular atrophy modifier implicates the SMN protein in SNARE complex assembly at neuromuscular synapses. *Neuron* 111 1423–1439. 10.1016/j.neuron.2023.02.004 36863345 PMC10164130

[B73] Kirisako T. Ichimura Y. Okada H. Kabeya Y. Mizushima N. Yoshimori T. (2000). The reversible modification regulates the membrane-binding state of Apg8/Aut7 essential for autophagy and the cytoplasm to vacuole targeting pathway. *J. Cell Biol.* 151 263–276. 10.1083/jcb.151.2.263 11038174 PMC2192639

[B74] Klionsky D. J. Abdel-Aziz A. M. Abdelfatah S. Abdellatif M. Abdoli A. Abel S. (2012). Guidelines for the use and interpretation of assays for monitoring autophagy. *Autophagy* 8 445–544. 22966490 10.4161/auto.19496 PMC3404883

[B75] Kolb S. J. Kissel J. T. (2015). Spinal muscular atrophy. *Neurol. Clin*. 33, 831–846. 10.1016/j.ncl.2015.07.004 26515624 PMC4628728

[B76] Koval E. D. Shaner C. Zhang P. du Maine X. Fischer K. Tay J. (2013). Method for widespread microRNA-155 inhibition prolongs survival in ALS-model mice. *Hum. Mol. Genet.* 22 4127–4135. 10.1093/hmg/ddt261 23740943 PMC3781640

[B77] Kumar A. V. Mills J. Lapierre L. R. (2022). Selective Autophagy Receptor p62/SQSTM1, a Pivotal Player in Stress and Aging. *Front. Cell Dev. Biol.* 10:793328. 10.3389/fcell.2022.793328 35237597 PMC8883344

[B78] Kye M. Niederst E. Wertz M. Gonçalves Ido C. Akten B. Dover K. (2014). SMN regulates axonal local translation via miR-183/mTOR pathway. *Hum. Mol. Genet.* 23 6318–6331. 10.1093/hmg/ddu350 25055867 PMC4271102

[B79] Lambert-Smith I. Saunders D. Yerbury J. (2020). The pivotal role of ubiquitin-activating enzyme E1 (UBA1) in neuronal health and neurodegeneration. *Int. J. Biochem. Cell Biol.* 123:105746. 10.1016/j.biocel.2020.105746 32315770

[B80] Lauria F. Bernabò P. Tebaldi T. Groen E. Perenthaler E. Maniscalco F. (2020). SMN-primed ribosomes modulate the translation of transcripts related to spinal muscular atrophy. *Nat. Cell Biol.* 22 1239–1251. 10.1038/s41556-020-00577-7 32958857 PMC7610479

[B81] Lee J. Shin J. Lee J. Choi E. (2015). Role of autophagy in the pathogenesis of amyotrophic lateral sclerosis. *Biochim. Biophys. Acta* 1852 2517–2524. 10.1016/j.bbadis.2015.08.005 26264610

[B82] Lee S. Sato Y. Nixon R. (2011). Lysosomal proteolysis inhibition selectively disrupts axonal transport of degradative organelles and causes an Alzheimer’s-like axonal dystrophy. *J. Neurosci.* 31 7817–7830. 10.1523/JNEUROSCI.6412-10.2011 21613495 PMC3351137

[B83] Levine B. Kroemer G. (2008). Autophagy in the pathogenesis of disease. *Cell* 132 27–42. 10.1016/j.cell.2007.12.018 18191218 PMC2696814

[B84] Li T. Feng Z. Wang Y. Zhang H. Li Q. Qin Y. (2020). antioncogenic effect of microRNA-206 on neck squamous cell carcinoma through inhibition of proliferation and promotion of apoptosis and autophagy. *Hum. Gene Ther.* 31 1260–1273. 10.1089/hum.2020.090 32900244

[B85] Li W. Li J. Bao J. (2012). Microautophagy: lesser-known self-eating. *Cell Mol. Life Sci.* 69 1125–1136. 10.1007/s00018-011-0865-5 22080117 PMC11114512

[B86] Lin S. Huang Y. Zhang L. Heng M. Ptácek L. Fu Y. (2013). MicroRNA-23a promotes myelination in the central nervous system. *Proc. Natl. Acad. Sci. U. S. A.* 110 17468–17473. 10.1073/pnas.1317182110 24101522 PMC3808585

[B87] Lörincz P. Juhász G. (2020). Autophagosome-Lysosome Fusion. *J. Mol. Biol.* 432 2462–2482. 10.1016/j.jmb.2019.10.028 31682838

[B88] Lorson C. Hahnen E. Androphy E. Wirth B. (1999). A single nucleotide in the SMN gene regulates splicing and is responsible for spinal muscular atrophy. *Proc. Natl. Acad. Sci. U. S. A.* 96 6307–6311. 10.1073/pnas.96.11.6307 10339583 PMC26877

[B89] Łusakowska A. Wójcik A. Frączek A. Aragon-Gawińska K. Potulska-Chromik A. Baranowski P. (2023). Long-term nusinersen treatment across a wide spectrum of spinal muscular atrophy severity: a real-world experience. *Orphanet. J. Rare Dis.* 18:230. 10.1186/s13023-023-02769-4 37542300 PMC10401775

[B90] MacLeod M. Taylor J. Lunt P. Mathew C. Robb S. (1999). Prenatal onset spinal muscular atrophy. *Eur. J. Paediatr. Neurol.* 3 65–72. 10.1053/ejpn.1999.0184 10700541

[B91] Magri F. Vanoli F. Corti S. (2018). miRNA in spinal muscular atrophy pathogenesis and therapy. *J. Cell Mol. Med.* 22 755–767. 10.1111/jcmm.13450 29160009 PMC5783860

[B92] Mariño G. Niso-Santano M. Baehrecke E. Kroemer G. (2014). Self-consumption: the interplay of autophagy and apoptosis. *Nat. Rev. Mol. Cell Biol.* 15 81–94. 10.1038/nrm3735 24401948 PMC3970201

[B93] Marquez R. T. Xu L. (2012). Bcl-2:Beclin 1 complex: multiple, mechanisms regulating autophagy/apoptosis toggle switch. *Am. J. Cancer Res.* 2 214–221. 22485198 PMC3304572

[B94] Martinet W. Agostinis P. Vanhoecke B. Dewaele M. De Meyer G. (2009). Autophagy in disease: A double-edged sword with therapeutic potential. *Clin. Sci.* 116 697–712. 10.1042/CS20080508 19323652

[B95] Mendell J. R. Al-Zaidy S. Shell R. Arnold W. Rodino-Klapac L. Prior T. (2017). Single-Dose Gene-Replacement Therapy for Spinal Muscular Atrophy. *N. Engl. J. Med.* 377 1713–1722. 10.1056/nejmoa1706198 29091557

[B96] Menzies F. M. Moreau K. Puri C. Renna M. Rubinsztein D. (2012). Measurement of Autophagic Activity in Mammalian Cells. *Curr. Protoc. Cell Biol.* 54 15. 10.1002/0471143030.cb1516s54 22422474

[B97] Meyer K. Ferraiuolo L. Schmelzer L. Braun L. McGovern V. Likhite S. (2015). Improving single injection CSF delivery of AAV9-mediated gene therapy for SMA: a dose-response study in mice and nonhuman primates. *Mol. Ther.* 23 477–487. 10.1038/mt.2014.210 25358252 PMC4351452

[B98] Miller N. Shi H. Zelikovich A. Ma Y. (2016). Motor neuron mitochondrial dysfunction in spinal muscular atrophy. *Hum. Mol. Genet.* 25 3395–3406. 10.1093/hmg/ddw262 27488123 PMC5179954

[B99] Miller R. Harrison D. Astle C. Fernandez E. Flurkey K. Han M. (2014). Rapamycin-mediated lifespan increase in mice is dose and sex dependent and metabolically distinct from dietary restriction. *Aging Cell* 13 468–477. 10.1111/acel.12194 24341993 PMC4032600

[B100] Mizushima N. Levine B. (2010). Autophagy in mammalian development and differentiation. *Nat. Cell Biol.* 12 823–830. 10.1038/ncb0910-823 20811354 PMC3127249

[B101] Mizushima N. Levine B. Cuervo A. Klionsky D. (2008). Autophagy fights disease through cellular self-digestion. *Nature* 451 1069–1075. 10.1038/nature06639 18305538 PMC2670399

[B102] Mizushima N. Yamamoto A. Matsui M. Yoshimori T. Ohsumi Y. (2004). In vivo analysis of autophagy in response to nutrient starvation using transgenic mice expressing a fluorescent autophagosome marker. *Mol. Biol Cell* 15 1101–1111. 10.1091/mbc.e03-09-0704 14699058 PMC363084

[B103] Mizushima N. Yoshimori T. Ohsumi Y. (2011). The role of Atg proteins in autophagosome formation. *Annu. Rev. Cell Dev. Biol.* 27 107–132. 10.1146/annurev-cellbio-092910-154005 21801009

[B104] Mohamud Y. Shi J. Qu J. Poon T. Xue Y. Deng H. (2018). Enteroviral Infection Inhibits Autophagic Flux via Disruption of the SNARE Complex to Enhance Viral Replication. *Cell Rep.* 22 3292–3303. 10.1016/j.celrep.2018.02.090 29562184

[B105] Monani U. Coovert D. Burghes A. (2000). Animal models of spinal muscular atrophy. *Hum. Mol. Genet.* 9 2451–2457. 10.1093/hmg/9.16.2451 11005801

[B106] Monani U. De Vivo D. (2014). Neurodegeneration in spinal muscular atrophy: from disease phenotype and animal models to therapeutic strategies and beyond. *Fut. Neurol.* 9 49–65. 10.2217/fnl.13.58 24648831 PMC3955729

[B107] Monani U. Lorson C. Parsons D. Prior T. Androphy E. Burghes A. (1999). A single nucleotide difference that alters splicing patterns distinguishes the SMA gene SMN1 from the copy gene SMN2. *Hum. Mol. Genet.* 8 1177–1183. 10.1093/hmg/8.7.1177 10369862

[B108] Montagnac G. Meas-Yedid V. Irondelle M. Castro-Castro A. Franco M. Shida T. (2013). αTAT1 catalyses microtubule acetylation at clathrin-coated pits. *Nature* 502 567–570. 10.1038/nature12571 24097348 PMC3970258

[B109] Mu Y. Yan X. Li D. Zhao D. Wang L. Wang X. (2018). NUPR1 maintains autolysosomal efflux by activating SNAP25 transcription in cancer cells. *Autophagy* 14 654–670. 10.1080/15548627.2017.1338556 29130426 PMC5959327

[B110] Nguyen D. Thombre R. Wang J. (2019). Autophagy as a common pathway in amyotrophic lateral sclerosis. *Neurosci. Lett.* 697 34–48. 10.1016/j.neulet.2018.04.006 29626651 PMC6170747

[B111] Nilsson P. Saido T. (2014). Dual roles for autophagy: degradation and secretion of Alzheimer’s disease Aβ peptide. *Bioessays* 36 570–578. 10.1002/bies.201400002 24711225 PMC4316186

[B112] Ning K. Drepper C. Valori C. Ahsan M. Wyles M. Higginbottom A. (2010). PTEN depletion rescues axonal growth defect and improves survival in SMN-deficient motor neurons. *Hum. Mol. Genet.* 19 3159–3168. 10.1093/hmg/ddq226 20525971

[B113] Nishio H. Niba E. Saito T. Okamoto K. Takeshima Y. Awano H. (2023). Spinal muscular atrophy: the past, present, and future of diagnosis and treatment. *Int. J. Mol. Sci.* 24 11939. 10.3390/ijms241511939 37569314 PMC10418635

[B114] Nixon R. (2013). The role of autophagy in neurodegenerative disease. *Nat. Med.* 19 983–997. 10.1038/nm.3232 23921753

[B115] Noda T. Ohsumi Y. (1998). Tor, a phosphatidylinositol kinase homologue, controls autophagy in yeast. *J. Biol. Chem.* 273 3963–3966. 10.1074/jbc.273.7.3963 9461583

[B116] Ogbonmide T. Rathore R. Rangrej S. Hutchinson S. Lewis M. Ojilere S. (2023). Gene Therapy for Spinal Muscular Atrophy (SMA): A Review of Current Challenges and Safety Considerations for Onasemnogene Abeparvovec (Zolgensma). *Cureus* 15 e36197. 10.7759/cureus.36197 37065340 PMC10104684

[B117] O’Hern P. Garcia E. L. Hao L. T. Hart A. C. Matera A. G. Beattie C. E. (2017). Nonmammalian animal models of spinal muscular atrophy. *Spinal Musc. Atrophy* 2017 221–239. 10.1016/B978-0-12-803685-3.00014-8

[B118] Ohsumi Y. (2001). Molecular dissection of autophagy: two ubiquitin-like systems. *Nat. Rev. Mol. Cell Biol.* 2 211–216. 10.1038/35056522 11265251

[B119] Oliván S. Calvo A. Rando A. Herrando-Grabulosa M. Manzano R. Zaragoza P. (2016). Neuroprotective effect of non-viral gene therapy treatment based on tetanus toxin c-fragment in a severe mouse model of spinal muscular atrophy. *Front. Mol. Neurosci.* 9:76. 10.3389/fnmol.2016.00076 27605908 PMC4995219

[B120] Oprea G. Kröber S. McWhorter M. Rossoll W. Müller S. Krawczak M. (2008). Plastin 3 is a protective modifier of autosomal recessive spinal muscular atrophy. *Science* 320 524–527. 10.1126/science.1155085 18440926 PMC4908855

[B121] Paik J. (2022). Risdiplam: A Review in Spinal Muscular Atrophy. *CNS Drugs* 36 401–410. 10.1007/s40263-022-00910-8 35284988

[B122] Pankiv S. Johansen T. (2010). FYCO1: linking autophagosomes to microtubule plus end-directing molecular motors. *Autophagy* 6 550–552. 10.4161/auto.6.4.11670 20364109

[B123] Passini M. Bu J. Richards A. Kinnecom C. Sardi S. Stanek L. (2011). Antisense oligonucleotides delivered to the mouse CNS ameliorate symptoms of severe spinal muscular atrophy. *Sci. Transl. Med.* 3:72ra18. 10.1126/scitranslmed.3001777 21368223 PMC3140425

[B124] Pattingre S. Tassa A. Qu X. Garuti R. Liang X. Mizushima N. (2005). Bcl-2 antiapoptotic proteins inhibit Beclin 1-dependent autophagy. *Cell* 122 927–939. 10.1016/j.cell.2005.07.002 16179260

[B125] Periyakaruppiah A. de la Fuente S. Arumugam S. Bahí N. Garcera A. Soler R. (2016). Autophagy modulators regulate survival motor neuron protein stability in motoneurons. *Exp. Neurol.* 283 287–297. 27373203 10.1016/j.expneurol.2016.06.032

[B126] Piras A. Schiaffino L. Boido M. Valsecchi V. Guglielmotto M. De Amicis E. (2017). Inhibition of autophagy delays motoneuron degeneration and extends lifespan in a mouse model of spinal muscular atrophy. *Cell Death Dis.* 8:3223. 10.1038/s41419-017-0086-4 29259166 PMC5870600

[B127] Poirier A. Weetall M. Heinig K. Bucheli F. Schoenlein K. Alsenz J. (2018). Risdiplam distributes and increases SMN protein in both the central nervous system and peripheral organs. *Pharmacol. Res. Perspect.* 6 e00447. 10.1002/prp2.447 30519476 PMC6262736

[B128] Powis R. Karyka E. Boyd P. Côme J. Jones R. Zheng Y. (2016). Systemic restoration of UBA1 ameliorates disease in spinal muscular atrophy. *JCI Insight* 1 e87908. 10.1172/jci.insight.87908 27699224 PMC5033939

[B129] Ragagnin A. Shadfar S. Vidal M. Jamali M. Atkin J. (2019). Motor Neuron Susceptibility in ALS/FTD. *Front. Neurosci.* 13:532. 10.3389/fnins.2019.00532 31316328 PMC6610326

[B130] Ramser J. Ahearn M. Lenski C. Yariz K. Hellebrand H. von Rhein M. (2008). Rare missense and synonymous variants in UBE1 are associated with X-linked infantile spinal muscular atrophy. *Am. J. Hum. Genet.* 82 188–193. 10.1016/j.ajhg.2007.09.009 18179898 PMC2253959

[B131] Ravikumar B. Vacher C. Berger Z. Davies J. Luo S. Oroz L. (2004). Inhibition of mTOR induces autophagy and reduces toxicity of polyglutamine expansions in fly and mouse models of Huntington disease. *Nat. Genet.* 36 585–595. 10.1038/ng1362 15146184

[B132] Riessland M. Kaczmarek A. Schneider S. Swoboda K. Löhr H. Bradler C. (2017). Neurocalcin delta suppression protects against spinal muscular atrophy in humans and across species by restoring impaired endocytosis. *Am. J. Hum. Genet.* 100 297–315. 10.1016/j.ajhg.2017.01.005 28132687 PMC5294679

[B133] Ripolone M. Ronchi D. Violano R. Vallejo D. Fagiolari G. Barca E. (2015). Impaired muscle mitochondrial biogenesis and myogenesis in spinal muscular atrophy. *JAMA Neurol.* 72 666–675. 10.1001/jamaneurol.2015.0178 25844556 PMC4944827

[B134] Rodriguez-Muela N. Parkhitko A. Grass T. Gibbs R. Norabuena E. Perrimon N. (2018). Blocking p62-dependent SMN degradation ameliorates spinal muscular atrophy disease phenotypes. *J. Clin. Invest.* 128 3008–3023. 10.1172/JCI95231 29672276 PMC6025996

[B135] Rose F. Mattis V. Rindt H. Lorson C. (2009). Delivery of recombinant follistatin lessens disease severity in a mouse model of spinal muscular atrophy. *Hum. Mol. Genet.* 18 997–1005. 10.1093/hmg/ddn426 19074460 PMC2649020

[B136] Rossoll W. Jablonka S. Andreassi C. Kröning A. Karle K. Monani U. (2003). Smn, the spinal muscular atrophy-determining gene product, modulates axon growth and localization of beta-actin mRNA in growth cones of motoneurons. *J. Cell Biol.* 163 801–812. 10.1083/jcb.200304128 14623865 PMC2173668

[B137] Roy S. (2021). Regulation of autophagy by miRNAs in human diseases. *Nucleus* 64 317–329. 10.1007/s13237-021-00378-9 34690368 PMC8520464

[B138] Rubinsztein D. Nixon R. (2010). Rapamycin induces autophagic flux in neurons. *Proc. Natl. Acad. Sci. U. S. A.* 107 E181. 10.1073/pnas.1014633107 21115811 PMC3000262

[B139] Sansa A. Hidalgo I. Miralles M. delaFuente S. Perez-Garcia M. Munell F. (2021). . Spinal Muscular Atrophy autophagy profile is tissue-dependent: differential regulation between muscle and motoneurons. *Acta Neuropathol. Commun.* 9:122. 10.1186/s40478-021-01223-5 34217376 PMC8254901

[B140] Sareen D. Ebert A. Heins B. McGivern J. Ornelas L. Svendsen C. (2012). Inhibition of apoptosis blocks human motor neuron cell death in a stem cell model of spinal muscular atrophy. *PLoS One* 7:e39113. 10.1371/journal.pone.0039113 22723941 PMC3378532

[B141] Sarkar S. Korolchuk V. Renna M. Imarisio S. Fleming A. Williams A. (2011). Complex inhibitory effects of nitric oxide on autophagy. *Mol. Cell.* 43 19–32. 10.1016/j.molcel.2011.04.029 21726807 PMC3149661

[B142] Sasaki S. (2011). Autophagy in spinal cord motor neurons in sporadic amyotrophic lateral sclerosis. *J. Neuropathol. Exp. Neurol.* 70 349–359. 10.1097/NEN.0b013e3182160690 21487309

[B143] Saxton R. Sabatini D. (2017). mTOR signaling in growth, metabolism, and disease. *Cell* 168 960–976. 10.1016/j.cell.2017.02.004 28283069 PMC5394987

[B144] Seto A. Beatty X. Lynch J. Hermreck M. Tetzlaff M. Duvic M. (2018). Cobomarsen, an oligonucleotide inhibitor of miR-155, co-ordinately regulates multiple survival pathways to reduce cellular proliferation and survival in cutaneous T-cell lymphoma. *Br. J. Haematol.* 183 428–444. 10.1111/bjh.15547 30125933

[B145] Shababi M. Lorson C. Rudnik-Schöneborn S. (2014). Spinal muscular atrophy: a motor neuron disorder or a multi-organ disease? *J. Anat.* 224 15–28. 10.1111/joa.12083 23876144 PMC3867883

[B146] Shang L. Chen S. Du F. Li S. Zhao L. Wang X. (2011). Nutrient starvation elicits an acute autophagic response mediated by Ulk1 dephosphorylation and its subsequent dissociation from AMPK. *Proc. Natl. Acad. Sci. U. S. A.* 108 4788–4793. 10.1073/pnas.1100844108 21383122 PMC3064373

[B147] Sharma M. McFarlane C. Kambadur R. Kukreti H. Bonala S. Srinivasan S. (2015). Myostatin: expanding horizons. *IUBMB Life* 67 589–600. 10.1002/iub.1392 26305594

[B148] Shimizu S. Honda S. Arakawa S. Yamaguchi H. (2014). Alternative macroautophagy and mitophagy. *Int. J. Biochem. Cell Biol.* 50 64–66. 10.1016/j.biocel.2014.02.016 24569119

[B149] Si X. Cao D. Chen J. Nie Y. Jiang Z. Chen M. (2018). miR-23a downregulation modulates the inflammatory response by targeting ATG12-mediated autophagy. *Mol. Med. Rep.* 18 1524–1530. 10.3892/mmr.2018.9081 29845275 PMC6072189

[B150] Singh N. Singh N. Androphy E. Singh R. (2006). Splicing of a critical exon of human Survival Motor Neuron is regulated by a unique silencer element located in the last intron. *Mol. Cell Biol.* 26 1333–1346. 10.1128/MCB.26.4.1333-1346.2006 16449646 PMC1367187

[B151] Singh R. Singh N. (2018). Mechanism of splicing regulation of spinal muscular atrophy genes. *Adv. Neurobiol.* 20 31–61. 10.1007/978-3-319-89689-2_2 29916015 PMC6026014

[B152] Sison S. Patitucci T. Seminary E. Villalon E. Lorson C. Ebert A. (2017). Astrocyte-produced miR-146a as a mediator of motor neuron loss in spinal muscular atrophy. *Hum. Mol. Genet.* 26 3409–3420. 10.1093/hmg/ddx230 28637335

[B153] Sivaramakrishnan M. McCarthy K. Campagne S. Huber S. Meier S. Augustin A. (2017). Binding to SMN2 pre-mRNA-protein complex elicits specificity for small molecule splicing modifiers. *Nat. Commun.* 8:1476. 10.1038/s41467-017-01559-4 29133793 PMC5684323

[B154] Song C. Guo J. Liu Y. Tang B. (2012). Autophagy and its comprehensive impact on ALS. *Int. J. Neurosci.* 122 695–703. 10.3109/00207454.2012.714430 22827270

[B155] Summers C. Valentine R. (2020). Acute Heat Exposure Alters Autophagy Signaling in C2C12 Myotubes. *Front. Physiol.* 10:1521. 10.3389/fphys.2019.01521 31969827 PMC6960406

[B156] Thomas N. Dubowitz V. (1994). The natural history of type I (severe) spinal muscular atrophy. *Neuromuscul. Disord.* 4 497–502. 10.1016/0960-8966(94)90090-6 7881295

[B157] Torres-Benito L. Neher M. Cano R. Ruiz R. Tabares L. (2011). SMN requirement for synaptic vesicle, active zone and microtubule postnatal organization in motor nerve terminals. *PLoS One.* 6:e26164. 10.1371/journal.pone.0026164 22022549 PMC3192162

[B158] Torres-Benito L. Schneider S. Rombo R. Ling K. Grysko V. Upadhyay A. (2019). NCALD antisense oligonucleotide therapy in addition to nusinersen further ameliorates spinal muscular atrophy in mice. *Am. J. Hum. Genet.* 105 221–230. 10.1016/j.ajhg.2019.05.008 31230718 PMC6612520

[B159] Ucar A. Gupta S. Fiedler J. Erikci E. Kardasinski M. Batkai S. (2012). The miRNA-212/132 family regulates both cardiac hypertrophy and cardiomyocyte autophagy. *Nat. Commun.* 3:1078. 10.1038/ncomms2090 23011132 PMC3657998

[B160] Valori C. Ning K. Wyles M. Mead R. Grierson A. Shaw P. (2010). Systemic delivery of scAAV9 expressing SMN prolongs survival in a model of spinal muscular atrophy. *Sci. Transl. Med.* 2:35ra42. 10.1126/scitranslmed.3000830 20538619

[B161] Valsecchi V. Anzilotti S. Serani A. Laudati G. Brancaccio P. Guida N. (2020). miR-206 Reduces the Severity of Motor Neuron Degeneration in the Facial Nuclei of the Brainstem in a Mouse Model of SMA. *Mol. Ther.* 28 1154–1166. 10.1016/j.ymthe.2020.01.013 32075715 PMC7132835

[B162] Valsecchi V. Boido M. De Amicis E. Piras A. Vercelli A. (2015). Expression of Muscle-Specific MiRNA 206 in the Progression of Disease in a Murine SMA Model. *PLoS One* 10:e0128560. 10.1371/journal.pone.0128560 26030275 PMC4450876

[B163] Verhaart I. Robertson A. Wilson I. Aartsma-Rus A. Cameron S. Jones C. (2017). Prevalence, incidence and carrier frequency of 5q-linked spinal muscular atrophy - a literature review. *Orphanet. J. Rare Dis.* 12:124. 10.1186/s13023-017-0671-8 28676062 PMC5496354

[B164] Wan J. Yang X. Ren Y. Li X. Zhu Y. Haddock A. (2019). Inhibition of miR-155 reduces impaired autophagy and improves prognosis in an experimental pancreatitis mouse model. *Cell Death Dis.* 10:303. 10.1038/s41419-019-1545-x 30944299 PMC6447551

[B165] Wang W. Lu G. Su X. Lyu H. Poon W. (2017). MicroRNA-182 Regulates Neurite Outgrowth Involving the PTEN/AKT Pathway. *Front. Cell Neurosci.* 11:96. 10.3389/fncel.2017.00096 28442995 PMC5385363

[B166] Wei Y. (2014). Autophagic induction of amyotrophic lateral sclerosis-linked Cu/Zn superoxide dismutase 1 G93A mutant in NSC34 cells. *Neural. Regen. Res.* 9 16–24. 10.4103/1673-5374.125325 25206739 PMC4146317

[B167] Williams A. Sarkar S. Cuddon P. Ttofi E. Saiki S. Siddiqi F. (2008). Novel targets for Huntington’s disease in an mTOR-independent autophagy pathway. *Nat. Chem. Biol.* 4 295–305. 10.1038/nchembio.79 18391949 PMC2635566

[B168] Wirth B. (2021). Spinal Muscular Atrophy: In the Challenge Lies a Solution. *Trends Neurosci.* 44 306–322. 10.1016/j.tins.2020.11.009 33423791

[B169] Wong E. Cuervo A. (2010). Autophagy gone awry in neurodegenerative diseases. *Nat. Neurosci.* 13 805–811. 10.1038/nn.2575 20581817 PMC4038747

[B170] Wu Y. T. Tan H. Shui G. Bauvy C. Huang Q. Wenk M. (2010). Dual role of 3-methyladenine in modulation of autophagy via different temporal patterns of inhibition on class I and III phosphoinositide 3-kinase. *J. Biol. Chem.* 285 10850–10861. 10.1074/jbc.M109.080796 20123989 PMC2856291

[B171] Xiong S. Mu T. Wang G. Jiang X. (2014). Mitochondria-mediated apoptosis in mammals. *Protein Cell* 5 737–749. 10.1007/s13238-014-0089-1 25073422 PMC4180462

[B172] Yamamoto A. Tagawa Y. Yoshimori T. Moriyama Y. Masaki R. Tashiro Y. (1998). Bafilomycin A1 prevents maturation of autophagic vacuoles by inhibiting fusion between autophagosomes and lysosomes in rat hepatoma cell line, H-4-II-E cells. *Cell Struct. Funct.* 23 33–42. 10.1247/csf.23.33 9639028

[B173] Yamashima T. (2013). Reconsider Alzheimer’s disease by the ‘calpain-cathepsin hypothesis’–a perspective review. *Prog. Neurobiol.* 105 1–23. 10.1016/j.pneurobio.2013.02.004 23499711

[B174] Yang J. Kim W. Kim D. (2023). Autophagy in cell survival and death. *Int. J. Mol. Sci.* 24:4744. 10.3390/ijms24054744 36902171 PMC10002575

[B175] Yanyan C. Yujin Q. Jinli B. Yuwei J. Hong W. Fang S. (2014). Correlation of PLS3 expression with disease severity in children with spinal muscular atrophy. *J. Hum. Genet.* 59 24–27. 10.1038/jhg.2013.111 24172247

[B176] Yorimitsu T. Klionsky D. (2005). Autophagy: molecular machinery for self-eating. *Cell Death Differ.* 12 1542–1552. 10.1038/sj.cdd.4401765 16247502 PMC1828868

[B177] Yuan Y. Zhang Y. Han L. Sun S. Shu Y. (2018). miR-183 inhibits autophagy and apoptosis in gastric cancer cells by targeting ultraviolet radiation resistance-associated gene. *Int. J. Mol. Med.* 42 3562–3570. 10.3892/ijmm.2018.3871 30221685

[B178] Yuva-Aydemir Y. Simkin A. Gascon E. Gao F. (2011). MicroRNA-9: functional evolution of a conserved small regulatory RNA. *RNA Biol.* 8 557–564. 10.4161/rna.8.4.16019 21697652 PMC3225974

[B179] Zerres K. Rudnik-Schöneborn S. (1995). Natural history in proximal spinal muscular atrophy. Clinical analysis of 445 patients and suggestions for a modification of existing classifications. *Arch. Neurol.* 52 518–523. 10.1001/archneur.1995.00540290108025 7733848

[B180] Zerres K. Rudnik-Schöneborn S. Forrest E. Lusakowska A. Borkowska J. Hausmanowa-Petrusewicz I. (1997). A collaborative study on the natural history of childhood and juvenile onset proximal spinal muscular atrophy (type II and III SMA): 569 patients. *J. Neurol. Sci.* 146 67–72. 10.1016/s0022-510x(96)00284-5 9077498

[B181] Zhang G. Wang J. Jia Y. Han R. Li P. Zhu D. (2015). MicroRNA-9 promotes the neuronal differentiation of rat bone marrow mesenchymal stem cells by activating autophagy. *Neural Regen. Res.* 10 314–320. 10.4103/1673-5374.143439 25883633 PMC4392682

[B182] Zhang Z. Pinto A. Wan L. Wang W. Berg M. Oliva I. (2013). Dysregulation of synaptogenesis genes antecedes motor neuron pathology in spinal muscular atrophy. *Proc. Natl. Acad. Sci. U. S. A.* 110 19348–19353. 10.1073/pnas.1319280110 24191055 PMC3845193

